# Reverse mutants of the catalytic 19 kDa mutant protein (nanoKAZ/nanoLuc) from *Oplophorus* luciferase with coelenterazine as preferred substrate

**DOI:** 10.1371/journal.pone.0272992

**Published:** 2022-09-21

**Authors:** Satoshi Inouye, Jun-ichi Sato, Yuiko Sahara-Miura, Yuri Tomabechi, Yuto Sumida, Shun-ichi Sekine, Mikako Shirouzu, Takamitsu Hosoya

**Affiliations:** 1 Yokohama Research Center, JNC Co., Kanazawa-ku, Yokohama, Japan; 2 Laboratory for Protein Functional and Structural Biology, RIKEN Center for Biosystems Dynamics Research (BDR), Tsurumi-ku, Yokohama, Japan; 3 Laboratory for Chemical Biology, RIKEN Center for Biosystems Dynamics Research (BDR), Chuo-ku, Kobe, Japan; 4 Laboratory for Transcription Structural Biology, RIKEN Center for Biosystems Dynamics Research (BDR), Tsurumi-ku, Yokohama, Japan; 5 Laboratory of Chemical Bioscience, Institute of Biomaterials and Bioengineering, Tokyo Medical and Dental University (TMDU), Chiyoda-ku, Tokyo, Japan; Kyoto University, JAPAN

## Abstract

Native *Oplophorus* luciferase (OpLase) and its catalytic 19 kDa protein (wild KAZ) show highest luminescence activity with coelenterazine (CTZ) among CTZ analogs. Mutated wild KAZ with 16 amino acid substitutions (nanoKAZ/nanoLuc) utilizes *bis*-coelenterazine (*bis*-CTZ) as the preferred substrate and exhibits over 10-fold higher maximum intensity than CTZ. To understand the substrate selectivity of nanoKAZ between CTZ and *bis*-CTZ, we prepared the reverse mutants of nanoKAZ by amino acid replacements with the original amino acid residue of wild KAZ. The reverse mutant with L18Q and V27L substitutions (QL-nanoKAZ) exhibited 2.6-fold higher maximum intensity with CTZ than that of nanoKAZ with *bis*-CTZ. The catalytic properties of QL-nanoKAZ including substrate specificity, luminescence spectrum, luminescence kinetics, luminescence products of CTZ, and luminescence inhibition by deaza-CTZ analogs were characterized and were compared with other CTZ-utilizing luciferases such as *Gaussia* and *Renilla* luciferases. Thus, QL-nanoKAZ with CTZ could be used as a potential reporter protein for various luminescence assay systems. Furthermore, the crystal structure of QL-nanoKAZ was determined at 1.70 Å resolution. The reverse mutation at the L18Q and V27L positions of α2-helix in nanoKAZ led to changes in the local structures of the α4-helix and the β6- and β7-sheets, and might enhance its binding affinity and oxidation efficiency with CTZ to emit light.

## Introduction

**Fig 1:**
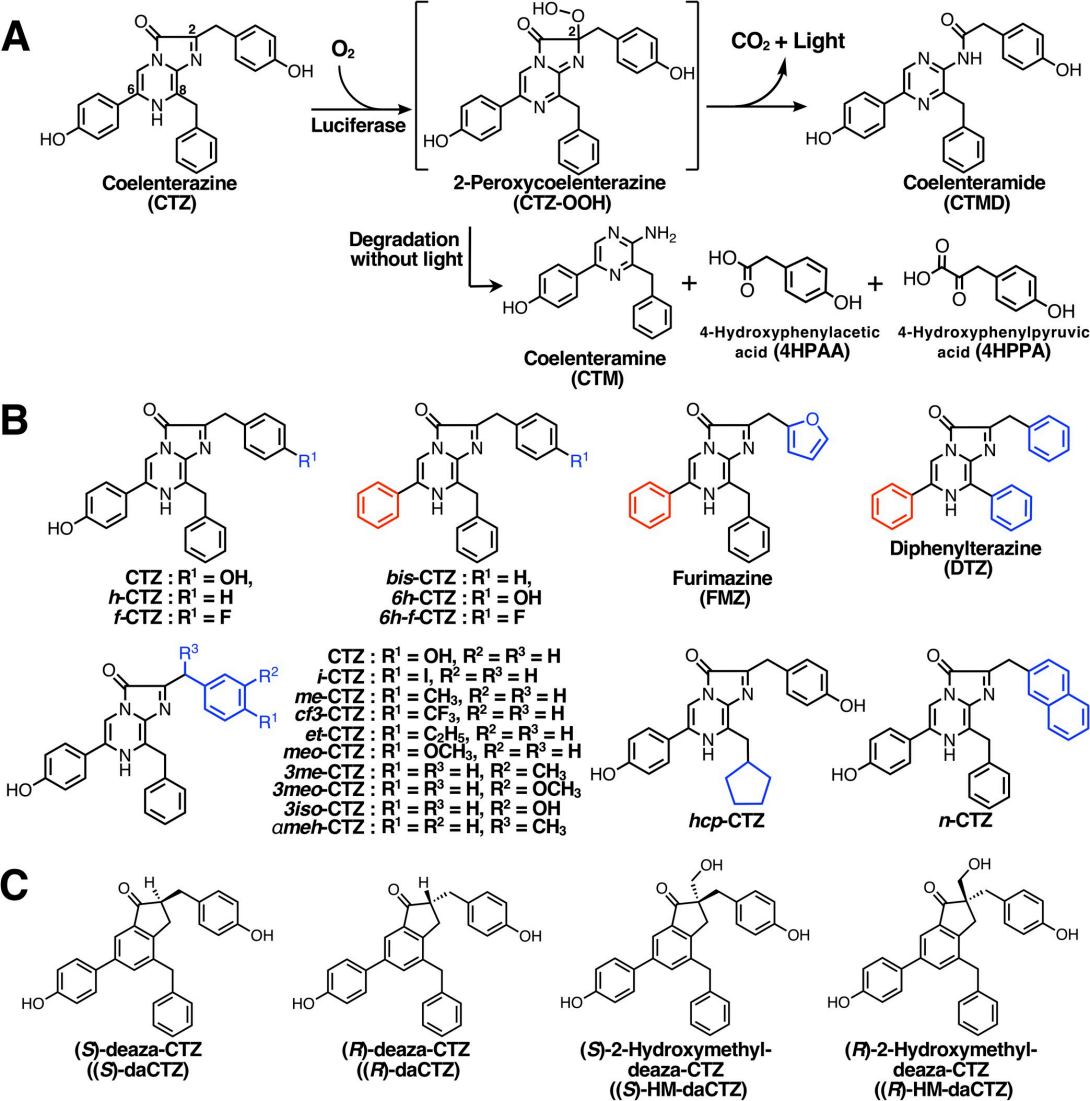
Luminescence reaction of coelenterazine (CTZ) catalyzed by the CTZ-utilizing luciferase and chemical structures of CTZ analogs and deaza-CTZ analogs. A. Oxidation process of CTZ with O_2_ by CTZ-utilizing luciferases and the degradation product of coelenteramine (CTM), 4-hydroxyphenylacetic acid (4HPAA), and 4-hydroxyphenylpyruvic acid (4HPPA) through 2-peroxycoelenterazine (CTZ-OOH). B. Chemical structures of C2- and C6-modified CTZ analogs. The C6-group of CTZ analogs was colored in red, and the C2- and C8-groups of CTZ analogs were colored in blue. C. Chemical structures of deaza-analogs for CTZ and CTZ-OOH as inhibitors.

*Oplophorus* luciferase (OpLase) is a secretory protein isolated from the deep-sea shrimp, *Oplophorus gracilirostris*, and catalyzes the oxidation of coelenterazine (CTZ, a luciferin) to emit blue light (λ_max_ = ~455 nm) [1,2] (**Fig 1A**). Molecular cloning of OpLase cDNA revealed that OpLase consists of 19 kDa and 35 kDa subunits with a molecular weight of 106 kDa [3]. The catalytic domain of OpLase was found in the 19 kDa subunit (wild KAZ) and was confirmed by protein expression in bacterial and mammalian cells [3,4]. However, the function of the 35 kDa subunit containing typical leucine-rich repeat sequences is still unknown [3].

Recently, the preferred human codon-optimized gene coding for nanoKAZ (mutant of wild KAZ with 16 amino acids substituted, GenBank accession no. AB823628) [5–8] with protein sequence identical to that of nanoLuc [9], was chemically synthesized and the functional nanoKAZ was successfully expressed in bacterial and mammalian cells. The protein of nanoKAZ was purified from *Escherichia coli* cells [5–8] and its crystal structure was determined (PDB ID: 5B0U). The structure of nanoKAZ consists of 11 antiparallel β-strands forming a β-barrel, capped by four short α-helices [10]. The central cavity of nanoKAZ was proposed to be a binding pocket of CTZ for the luminescence reaction [10]. However, the interacting moieties between CTZ and the amino acid residue(s) in nanoKAZ could not be identified.

Previously, we reported that OpLase shows broad substrate specificity for CTZ analogs [11–13], compared to other CTZ-utilizing luciferases including *Renilla* luciferase (RLase) [11], *Gaussia* luciferase (GLase) [13], and *Periphylla* luciferase [14]. Interestingly, only OpLase can efficiently use both CTZ and *bis*-coelenterazine (*bis*-CTZ) as substrates [11,12] (**Fig 1B**), and wild KAZ also showed similar substrate specificity [5,11]. In contrast to OpLase and wild KAZ, nanoKAZ could use *bis*-CTZ as the preferred substrate than CTZ with over 10-fold higher activity [5]. As previously reported [5], furimazine (FMZ) [9] with a furanylmethyl group instead of a benzyl group at the C2-position of *bis*-CTZ showed only half luminescence activity with *bis*-CTZ and is unsuitable substrate for nanoKAZ/nanoLuc.

To understand the differences in substrate specificity between wild KAZ and nanoKAZ, we first prepared the chimeric proteins of wild KAZ and nanoKAZ, and then prepared reverse mutants of nanoKAZ by replacing the original amino acid residues of wild KAZ at the same position (referred to as “reverse mutant”). The substrate specificities for reverse mutants toward CTZ and its analogs were investigated and the reverse nanoKAZ mutant with L18Q and V27L substitutions (assigned “QL-nanoKAZ or QL-nK”) could utilize CTZ as the preferred substrate with over 2.5-fold higher activity compared with *bis*-CTZ. The luminescence properties of QL-nanoKAZ were characterized and compared with other CTZ-utilizing luciferases, including native OpLase [2], nanoKAZ [5], nanoKAZ mutant with three amino acids substitutions (SNH-nanoKAZ = teLuc) [15], GLase [16], RLase [17], and RLase mutant with red-shifted luminescence spectrum (RLase-547 = RLuc8.6–547) [18]. Furthermore, the crystal structure of QL-nanoKAZ was determined (PDB ID: 7VSX) and the substrate recognition of QL-nanoKAZ for CTZ was discussed based on the results of substrate specificity for CTZ analogs and luminescence inhibition with deaza-coelenterazine analogs [19].

## Materials and methods

### Materials

The sources of chemicals were as follows: isopropyl β-thiogalactopyranoside (IPTG), ethylenediaminetetraacetic acid disodium salt (EDTA•2Na), (±)-dithiothreitol (DTT), and imidazole (Wako Pure Chemicals, Osaka, Japan); chelate Sepharose Fast Flow (GE-Healthcare Bio-Science, Piscataway, NJ, USA); coelenterazine (CTZ), coelenteramine (CTM), coelenteramide (CTMD), *h-*coelenterazine (*h*-CTZ), *bis*-coelenterazine (*bis*-CTZ), and *f-*coelenterazine (*f*-CTZ) (JNC Co., Tokyo, Japan). *6h-*Coelenterazine (*6h-*CTZ), *6h-f-*coelenterazine (*6h-f*-CTZ), and furimazine (FMZ) were synthesized as previously reported [5] (**Fig 1B**). The syntheses of the C2-modified CTZ analogs [20] (**Fig 1B**) and deaza-CTZ analogs [19] (**Fig 1C**) were described in our previous reports. Oligonucleotides used for site-directed mutagenesis and the synthetic gene of SNH-nanoKAZ (a nanoKAZ mutant with three amino acid substitutions; D19S, D85N, and C169H) which has the identical amino acid sequence to teLuc [15]), were obtained from Eurofins Genomics (Tokyo, Japan). Native OpLase (Lot. 20011221-red labeled fraction, >90% purity) was kindly provided by Dr. Osamu Shimomura (Marine Biology Laboratory, Woods Hole, MA, USA).

### Recombinant luciferases

Recombinant histidine-tagged luciferases for RLase [17], RLase-547 (a mutant of RLase with an emission peak at 547 nm, known as RLuc8.6–547 [18]), nanoKAZ (a mutant with 16 amino acids substituted in wild KAZ [3] obtained from the preferred human-codon optimized gene [5]), and SNH-nanoKAZ [15] were expressed in the cytoplasm of *E*. *coli* strain BL21 (Novagen, Madison, WI) using a cold induction system with the expression vectors of pCold-RL [13], pCold-RL 547 [13], pCold-dnKAZ [10], pCold-QL-nanoKAZ (this work), and pCold-SNH-nanoKAZ (this work), and purified using a Ni-chelate column, as previously described [10,13]. Recombinant GLase with the histidine-tagged sequence at the carboxyl terminus was stably expressed into the culture medium from a dihydrofolate reductase-deficient Chinese hamster ovary cells (CHO-K1/*dhfr*^*−*^cells) possessing a pcDNA3-hGL-H vector and was purified from the serum-containing cultured medium using a Ni-chelate column [21].

### Preparation of mutated genes for nanoKAZ

**Fig 2:**
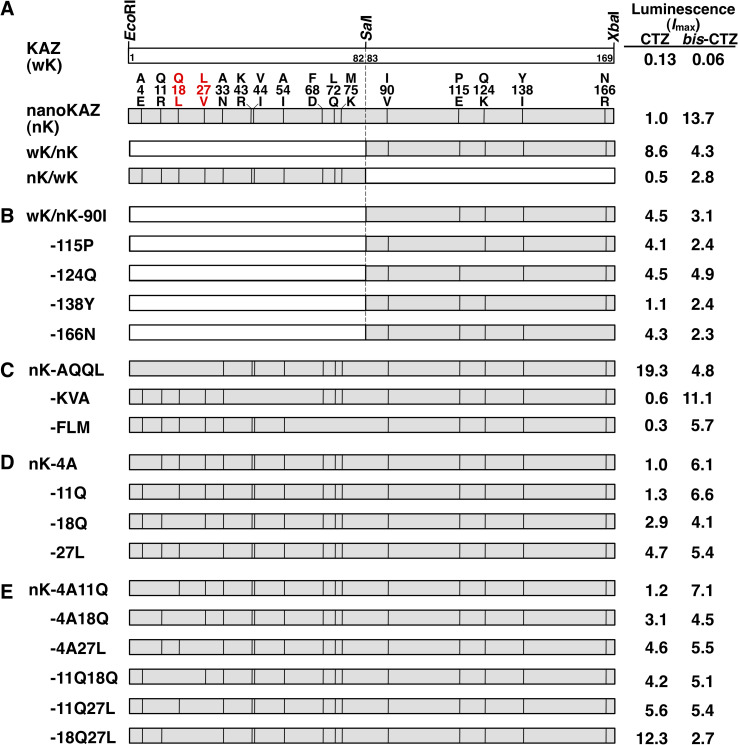
Schematic representation of the reverse mutants for nanoKAZ (nK) and their luminescence activities using coelenterazine (CTZ) and *bis*-coelenterazine (*bis*-CTZ) as substrates. A. Chimeric proteins between wild KAZ (wK) and nanoKAZ (nK). wK/nK, 1–82 aa of wK and 83–169 aa of nK; nK/wK, 1–82 aa of nK and 83–169 aa of wK. B. Reverse mutants of wK/nK chimera with a single amino acid substitution at the carboxyl terminal region of nanoKAZ (83–169 aa). C. Reverse mutants of nanoKAZ with three or four amino acid substitutions at the amino- terminal region of nanoKAZ (1–82 aa). D. Reverse mutants of nanoKAZ with a single amino acid substitution at the amino-terminal region of nanoKAZ (1–27 aa). E. Reverse mutants of nanoKAZ with double amino acid substitutions at the amino-terminal region of nanoKAZ (1–27 aa).

The chimeric genes for wild KAZ (wK), nanoKAZ (nK), and their reverse mutants were prepared as follows:

i) The chimeric genes for wK/nK chimera and nK/wK chimera were prepared by replacing the *Eco*RI-*Sal*I fragment (amino acid residues 1–82) or *Sal*I-*Xba*I fragment (amino acid residues 83–169) of wild KAZ with the corresponding fragments of nanoKAZ, respectively (**Fig 2A**).

ii) The reverse mutant genes of wK/nK chimera (**Fig 2B**) and nanoKAZ (**Fig 2C**–**2E**) were prepared by site-directed mutagenesis using the overlap extension polymerase chain reaction (PCR) procedures [22] with the specific primers (**S1 Table**). The nucleotide sequences of mutated genes were confirmed by DNA sequencing (Eurofins Genomics).

### Expression of mutated proteins in bacterial cells

The cold-inducible expression vector of pCold-ZZ-P-X under the control of the cold shock protein A promoter and the *lac* operator in *E*. *coli* cells [23,24] was used to express as a fused protein of the ZZ domain, which is the synthetic IgG-binding domain of staphylococcal protein A and serves as a soluble partner of target proteins [23,24]. The vector consists of a histidine tag sequence for Ni-chelate affinity chromatography, the cleavage sequence of human rhinovirus 3C protease between the ZZ domain and a target protein, followed by the multiple cloning sites [24]. The *Eco*RI-*Xba*I fragment of the reverse mutated gene was inserted into the *Eco*RI-*Xba*I site of a pCold-ZZ-P-X vector to give the corresponding expression vector.

**Fig 3:**
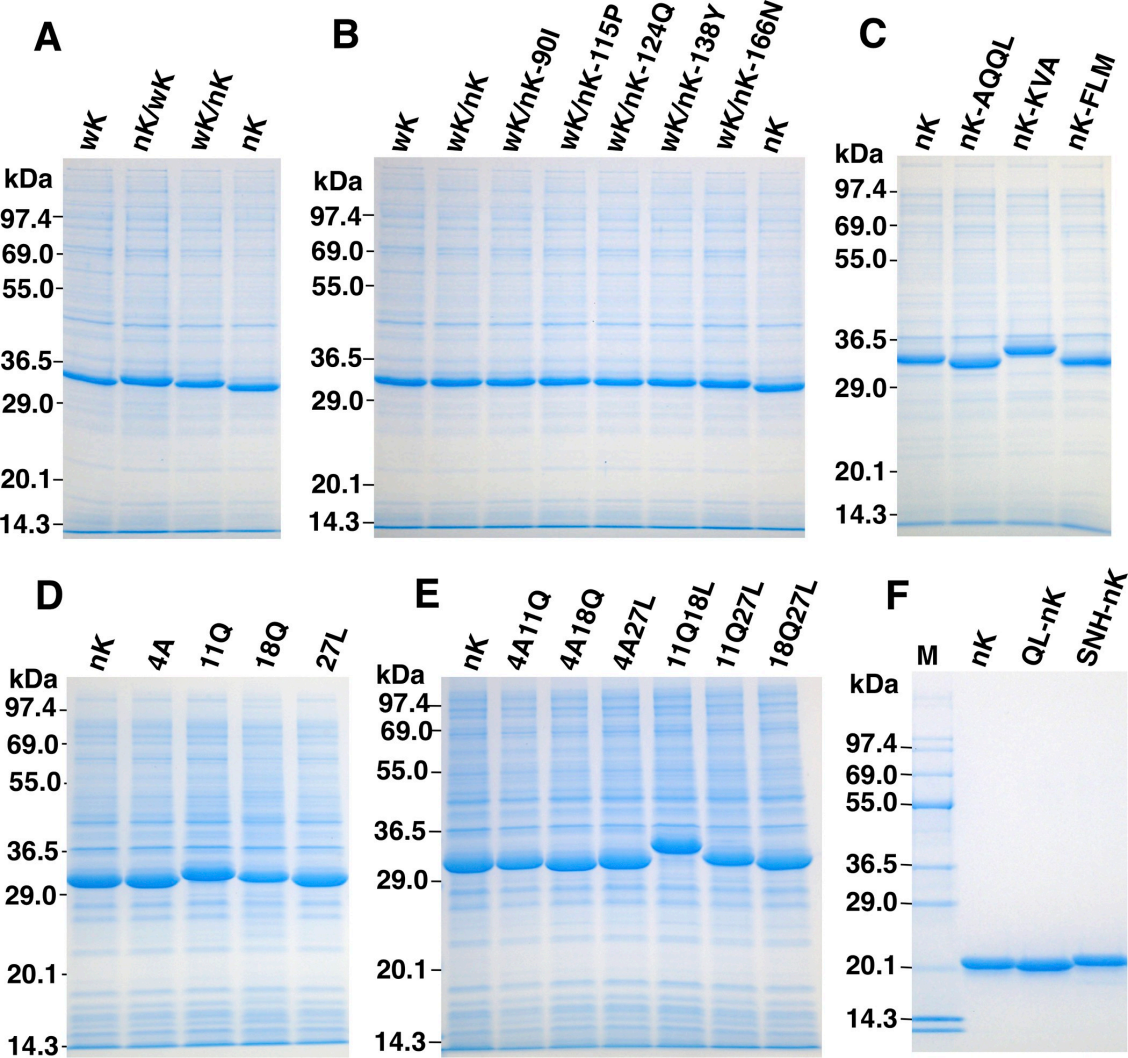
SDS-PAGE analyses of the soluble fractions of the reverse mutants expressed in *E*. *coli* cells using a pCold-ZZ-P-X vector (A-E) and the purified nanoKAZ, QL-nanoKAZ, and SNH-nanoKAZ from *E*. *coli* cells (F). A-E, the soluble fractions of mutant proteins obtained from *E*. *coli* cells by centrifugation at 12,000 × *g* for 3 min. Panels A-E correspond to those in Fig 2. The soluble fraction (5 μL) corresponded to 10 μL of the cultured cells was applied on a lane. F, purified nanoKAZ (nK), QL-nanoKAZ (QL-nK), and SNH-nanoKAZ (SNH-nK) from *E*. *coli* cells using a Ni-chelate column. Each luciferase (10 μg protein) was applied. M, molecular weight markers. The numbers on the left margin represent the molecular weight of marker proteins (TEFCO): Phosphorylase b (97.4 kDa), bovine serum albumin (69.0 kDa), glutamic dehydrogenase (55.0 kDa), lactic dehydrogenase (36.5 kDa), carbonic anhydrase (29.0 kDa), trypsin inhibitor (20.1 kDa), and lysozyme (14.3 kDa).

To determine luminescence activity of reverse mutants, the seed culture of *E*. *coli* strain BL21 possessing each expression vector was grown in 5 mL of Luria-Bertani broth (LB broth) containing ampicillin (50 μg/mL) at 37°C for 18 h using a TAITEC model BR-3000LF shaker (Tokyo, Japan) with reciprocal shaking (130 rpm). After transferring 0.1 mL of the seed culture to 10 mL of LB broth, the bacterial cells were cultured at 37°C for 3 h and then cooled on an ice bath over 30 min. To induce protein expression, 1 M IPTG was added to the culture medium at a final concentration of 1 mM and the bacterial cells were cultured at 15°C for 20 h. After collecting 1 mL of cultured cells using a TOMY model MCX-150 micro-centrifuge (Tokyo, Japan) at 8,000 rpm for 30 s, cells were suspended in 0.5 mL of 50 mM Tris-HCl (pH 7.6) and disrupted by sonication using a Branson model 250 sonifier (Danbury, CT) for 5 s in an ice-water bath. The soluble fraction of cell extracts obtained by centrifugation at 12,000 × *g* for 3 min was used for determining the luminescence activity and 5 μL was subjected to SDS-PAGE analysis (**Fig 3**).

### Expression and purification of QL-nanoKAZ and SNH-nanoKAZ

For purification of QL-nanoKAZ, the *Eco*RI-*Xba*I fragment of 18Q27L-nanoKAZ gene was replaced with nanoKAZ gene in pCold-dnKAZ [10] to give an expression vector, pCold-QL-nanoKAZ. The expressed QL-nanoKAZ consisted of 191 amino acid residues having the amino-terminal sequence of MNHKVHHHHHHMELGTLEGSEF (histidine-tag sequence underlined). The purification was carried out using Ni-chelate column chromatography [5,10]. Briefly, the seed culture (10 mL) in LB broth was transferred to 400 mL of LB broth containing 100 μL of antifoam (Disform CE475, NOF Co., Tokyo, Japan) in a 2-L Sakaguchi flask and cultured with reciprocal shaking (130 rpm) at 37°C for 3 h. The absorbance at 660 nm measured by TAITEC model mini photo 518R was 0.97 and reached 1.22 after 18 h at 15°C by the addition of IPTG to a final concentration of 0.2 mM. The harvested cells from 800 mL of culture medium (wet weight, 5.1 g) by using a Hitachi model CR20GIII high-speed refrigerated centrifuge (Tokyo, Japan) at 5,000 rpm for 5 min were suspended in 60 mL of 50 mM Tris-HCl (pH 7.6) and disrupted by sonication three times for 3 min in an ice-water bath. The soluble fractions obtained by centrifugation at 10,000 rpm for 20 min were applied on a Ni-chelate column (ø2.5 × 5 cm) at room temperature. After washing with 200 mL of 50 mM Tris-HCl (pH 7.6), the fractions of QL-nanoKAZ containing 154.2 mg proteins were obtained by eluting with 0.1 M imidazole (Wako Pure Chemicals) in 50 mM Tris-HCl (pH 7.6). For further purification, a portion of the eluted QL-nanoKAZ fractions (98.8 mg protein) from the 1st Ni-chelate column was diluted to 200 mL with 50 mM Tris-HCl (pH 7.6) containing 2 M NaCl and was applied on the 2nd Ni-chelate column (ø1.5 × 6 cm) and the proteins were eluted with 0.1 M imidazole. The highly purified QL-nanoKAZ (91.8 mg protein) was obtained (**Fig 3F** and **S2 Table**) and used as a protein source for crystal structure analysis.

For purification of SNH-nanoKAZ, the *Eco*RI-*Xba*I fragment of for SNH-nanoKAZ gene was chemically synthesized (Eurofine Genomics) and was replaced with the *Eco*RI-*Xba*I fragment of nanoKAZ gene in pCold-dnKAZ [10] to give an expression vector, pCold-SNH-nanoKAZ. The purification procedures were essentially the same as that of QL-nanoKAZ, and the yield was 135.6 mg protein from 800 mL of cultured cells (**Fig 3F**).

### Expression of luciferase genes in mammalian cells

For the secretory expression of wild KAZ mutants in mammalian cells, a pcDNA3-GLsp vector that is a derivative of pcDNA3 (Invitrogen, Carlsbad, CA) having the signal peptide sequence of GLase for secretion was used, as previously described [5]. The expression vectors were constructed as follows. The fragment of the mutated KAZ gene obtained by PCR procedures was inserted into the *Hin*dIII-*Xba*I sites of pcDNA3 or the *Eco*RI/*Xba*I site of pcDNA3-GLsp [5] to give pcDNA3-dnKAZ, pcDNA3-AQQL-nK (this work), and pcDNA3-QL-nK (this work) for expression in the cytoplasm, or pcDNA3-GLsp-dnKAZ [6], pcDNA3-GLsp-QL-nK (this work), pcDNA3-GLsp-SNH-nK (this work), and pcDNA3-GLsp-EpGLuc [25,26] for secretory expression. These vectors had the identical nucleotide sequence between the promoter and the initial methionine codon in the same vector [25,26]. For transient expression in Chinese hamster ovary-K1 (CHO-K1) cells, CHO-K1 cells (1 × 10^5^ cells) cultured in 2 mL of Ham’s F-12 medium (Wako Pure Chemicals) containing 10% (v/v) heat-inactivated fetal calf serum (Biowest, France) without antibiotics were seeded in a 6-well plate (*n* = 4) at 37°C in a humidified atmosphere of 5% CO_2_ for 24 h. For transfection, the mixture of the expression vector (1 μg) and FuGENE HD (3 μL, Promega) in 100 μL of serum-free Ham’s F-12 medium was added to the cells. After further incubation for 24 h, the culture medium was recovered and the cells were washed three times with 2 mL of PBS (D-PBS (–), Wako Pure Chemicals), and then lysed in 400 μL of lysis buffer (2 mM EDTA, 1% Triton-X 100 (Sigma) and 10% glycerol (Wako Pure Chemicals) in PBS), for 15 min at room temperature (24–26°C). The culture medium and cell extracts (2 μL) were used for luminescent assays (*n* = 2) [25,26].

### Assay for luminescence activity

The luminescence activity was determined using an ATTO (Tokyo, Japan) AB2200 luminometer (Ver.2.07, rev4.21) in the presence of a 0.23% neutral density filter (determined by using a laser at 544 nm) or an ATTO AB2270 luminometer with an F2-cut filter (HOYA, R62). Under these measurement conditions, the luminescence intensity of AB2270 showed 1.8-fold higher than that of AB2200 using aequorin as a light standard [27].

The reaction mixture (100 μL) contained CTZ or CTZ analog (1 μg/μL dissolved in ethanol) in 30 mM Tris-HCl (pH 7.6)–10 mM EDTA, and the luminescence reaction was initiated by the addition of 1–5 μL of luciferase solutions. The luminescence intensity was recorded in 0.1 s-intervals for 10–60 s using an AB2200 or AB 2270 luminometer. The maximum intensity of luminescence (*I*_max_) and the integrated luminescence intensity (*Int*.) were shown as relative light units (rlu).

### Bioluminescence spectral analysis

Bioluminescence spectra were measured with a fluorescence spectrophotometer FP-6500 (Jasco, Tokyo, Japan) at 24°C with the excitation light source turned off (emission bandwidth, 20 nm; sensitivity, medium; response, 0.5 s; scan speed, 1000 nm/min). The corrected bioluminescence spectra were obtained by the manufacturer’s protocol. The reaction mixture (1 mL) in a quartz cuvette contained 5 μg of CTZ or CTZ analog (dissolved in 5 μL of ethanol) in 30 mM Tris-HCl (pH 7.6)–10 mM EDTA, and the luminescence reaction was initiated by adding 5 μL of the purified luciferase (1.0 μg protein) to the reaction mixture.

### Protein analysis

SDS-PAGE analysis was carried out under reducing conditions using a 12% separation gel (TEFCO, Tokyo, Japan) and the gel was stained with a colloidal CBB staining kit (TEFCO). The protein concentrations of nanoKAZ, QL-nanoKAZ, SNH-nanoKAZ, GLase, and aequorin were determined by amino acid composition analysis, as previously described [27], and other luciferases were determined by the dye-binding method using a commercially available kit (Bio-Rad, Richmond, CA, USA) and bovine serum albumin as a standard (Pierce, Rockford, IL, USA).

### HPLC analysis of the reaction products of CTZ by the CTZ-utilizing luciferase

The reaction mixture containing each CTZ-utilizing luciferase (5 μg/5 μL) and CTZ (2 μg = 473 pmol/μL dissolved in ethanol) in 100 μL of 50 mM Tris-HCl (pH 7.6) was incubated at 25°C for 2 h and then was added to 100 μL of 6 M guanidine-HCl in H_2_O (pH 7.3) (Wako Pure Chemicals), and was extracted with 500 μL of diethyl ether using a vortex mixer for 15 s. After centrifugation at 10,000 rpm for 3 min at room temperature, 450 μL of ether layer was recovered and was dried down *in vacuo*. The resultant residues were dissolved in 100 μL of ethanol, showing purple-blue fluorescence under a 365 nm lamp, and 10 μL of the solution was subjected to HPLC analysis for CTZ, CTMD, CTM, CTO, and dCTZ [19,28]. An Agilent (CA, USA) 1200 series HPLC system was used under the following conditions: column, Wakosil 5C4 (ø4.6 mm × 250 mm); eluting solvent, CH_3_CN/H_2_O containing 0.1% trifluoroacetic acid; gradient elution, 40% CH_3_CN for 10 min, 40–50% CH_3_CN for 20 min, 50–80% CH_3_CN for 10 min, and 80% CH_3_CN for 10 min; flow rate, 0.5 mL/min; column temperature, 25°C; and detector, 225, 280, 330 and 450 nm using a diode array detector.

### Crystallization, data collection, and structure determination

The eluted fractions of QL-nanoKAZ (50 mg protein) from the Ni-chelate column were diluted twice with the buffer (20 mM Tris-HCl (pH 8.5) and 2 mM DTT) and then loaded on a HiTarp Q HP column (Cytiva, MA, USA). The fractions containing QL-nanoKAZ were eluted at 200 mM NaCl by a linear gradient from 50 mM to 400 mM of NaCl and concentrated by an Amicon Ultra centrifugal filter unit (MWCO 10,000, Merck-Millipore, Billerica, MA, USA), and separated by a HiLoad 16/60 Superdex 75 column (Cytiva) equilibrated with 20 mM Tris-HCl (pH 8.0) containing 150 mM NaCl and 2 mM DTT. The fractions of QL-nanoKAZ were concentrated to a concentration of 20 mg/mL by a centrifugal filter unit and stored at –80°C.

The crystallization was performed by the method of sitting drop vapor diffusion. The crystals were grown in a mixture of 0.2 μL of QL-nanoKAZ and 0.2 μL of the precipitant solution (100 mM MES (pH 6.5) and 1.9 M MgSO_4_) at 20°C. After a few days of incubation for equilibration against the precipitant solution, the crystals with dimensions of 250 μm × 250 μm × 20 μm were obtained **(S1 Fig)**. The crystals were cryoprotected in the reservoir solution supplemented with 25% (v/v) glycerol before flash-cooling in liquid nitrogen. An X-ray diffraction dataset was collected to 1.70 Å at a wavelength of 1.0 Å on beamline BL26B2 at SPring-8 [29]. The diffraction data were processed using the XDS programs [30] and the structure was solved by molecular replacement using the Phaser program [31] from the PHENIX programs [32], with the nanoKAZ coordinates (PDB ID: 5B0U) as the search model. The structural model was built into the electron density map using COOT [33] and refined using the PHENIX program. All structure figures were prepared using PyMOL (http://pymol.sourceforge.net/).

## Results and discussion

### Soluble expression of nanoKAZ mutants in bacterial cells

Previous studies have reported that wild KAZ and its mutated proteins were mainly expressed as inclusion bodies in *E*. *coli* cells [3,4]. To express soluble KAZ mutants in *E*. *coli* cells [5–7], the mutated proteins were expressed as the fusion protein with the ZZ domain of staphylococcal protein A using the cold-induced expression vector, pCold-ZZ-P-X [24]. The chimeric genes between wild KAZ and nanoKAZ (**Fig 2A**) and the reverse mutated genes of nanoKAZ were prepared using PCR procedures (**Fig 2B**–**2E**), and the mutated proteins fused to the ZZ domain were all successfully expressed as soluble form in *E*. *coli* cells.

The soluble fraction obtained by centrifugation was analyzed by SDS-PAGE (**Fig 3A**–3E), suggesting that the expression levels of each protein were not significantly different among them under the same culture conditions. Thus, the soluble fractions were used as luciferase sources for the luminescence assay without further purification. The luminescence activities of *I*_max_ and *Int*. 60 s were used as tentative indicators for substrate specificity.

### Substrate selectivity of chimera mutants and reverse mutants

To characterize the substrate specificities of the wK/nK chimera consisting of 1–82 aa of wild KAZ (wK) and 83–169 aa of nanoKAZ (nK) and the nK/wK chimera consisting of 1–82 aa of nK and 83–169 aa of wK, these were expressed in *E*. *coli* cells and the soluble fraction was used for assays (**Fig 2A**). As shown in **Table 1A**, the soluble fraction of nanoKAZ fused to the ZZ domain showed the following luminescence activities of *I*_max_: CTZ (1.0), *h-*CTZ (17.4), *6h-*CTZ (0.7), *bis*-CTZ (13.7), *f-*CTZ (15.3), *6h-f-*CTZ (10.5), and FMZ (6.3), similar to that of purified nanoKAZ protein [5].

**Table 1:** Luminescence activities of chimeric proteins among wild KAZ, nanoKAZ, and their reverse mutants of nanoKAZ using coelenterazine (CTZ) and CTZ analogs as a substrate.

nanoKAZ mutant ^a^ (ZZ domain fused protein)	Relative luminescence activity ^b^, *I*_max_ (*Int*. 60 ^c^)													
	CTZ		*h*-CTZ		*6h*-CTZ		*bis*-CTZ		*f*-CTZ		*6h-f-*CTZ		FMZ	
***A***. nanoKAZ (nK)	1.0 ^d^	(1.0) ^e^	17.4	(12.9)	0.7	(0.6)	13.7	(13.5)	15.3	(12.8)	10.5	(9.5)	6.3	(6.0)
KAZ (wK)	0.13	(0.14)	0.16	(0.13)	0.01	(0.02)	0.06	(0.10)	0.13	(0.12)	0.04	(0.06)	0.03	(0.05)
wK/nK	8.6	(7.9) ↑	8.6	(4.6)	1.2	(1.0)	4.3	(3.4)	6.4	(4.5)	5.5	(4.7)	3.5	(4.1)
nK/wK	0.5	(0.6)	5.3	(4.4)	0.2	(0.2)	2.8	(2.8)	4.5	(3.5)	2.3	(1.5)	2.0	(1.5)
***B***. wK/nK-90I	4.5	(5.0)↑	5.5	(3.1)	0.7	(0.8)	3.1	(2.9)	4.2	(3.0)	3.1	(3.2)	2.3	(2.1)
wK/nK-115P	4.1	(3.7)↑	3.6	(2.4)	0.8	(0.9)	2.4	(2.7)	2.9	(2.0)	1.8	(1.5)	1.7	(1.6)
wK/nK-124Q	4.5	(4.5)↑	9.3	(5.8)	6.2	(6.1) ↑	4.9	(4.7)	8.6	(5.6)	5.4	(4.9)	2.4	(1.5)
wK/nK-138Y	1.1	(1.2)	4.6	(3.1)	0.2	(0.4)	2.4	(2.3)	3.9	(2.5)	2.3	(1.4)	1.5	(1.3)
wK/nK-166N	4.3	(5.0)↑	5.7	(3.9)	0.6	(0.9)	2.3	(2.5)	5.1	(3.8)	1.8	(1.2)	2.2	(1.7)
***C***. nanoKAZ (nK)	1.0 ^f^	(1.0) ^g^	14.2	(7.4)	0.6	(0.4)	9.0	(6.3)	20.2	(10.0)	7.9	(4.6)	4.8	(3.7)
nK-AQQL	19.3	(11.6↑	28.9	(10.5)	5.4	(3.9) ↑	4.8	(4.9)	21.0	(8.0)	4.6	(5.0)	2.3	(2.6)
nK-KVA	0.6	(0.6)	9.1	(4.7)	0.1	(3.9)	11.1	(7.5)	17.4	(7.2)	11.9	(7.7)	5.4	(3.6)
nK-FLM	0.3	(0.3)	9.5	(6.5)	0.2	(0.1)	5.7	(3.2)	10.9	(5.9)	9.0	(4.7)	3.0	(2.3)

^a^ The mutated protein fused to ZZ domain was expressed in *E*. *coli* cells using a pCold-ZZ-P-X vector [24].

^b^ The luminescence activity (*n* = 2) was determined using an AB2200 luminometer with a 0.23% neutral density filter and shown as the relative intensity to that of nanoKAZ (nK) with CTZ. The experiment ***C*** was performed separately from the experiments ***A*** and ***B***.

^c^ Integration for 60 s in 0.1 s-intervals.

^d^ *I*_max_ = 2.8 × 10^5^ rlu/0.1 s.

^e^ *Int*. 60 s = 8.3 × 10^7^ rlu/60 s.

^f^ *I*_max_ = 1.2 × 10^4^ rlu/0.1 s.

^g^ *Int*. 60 s = 5.0 × 10^6^ rlu/60 s.

^h^ Vertical arrows (↑) indicate over 50% increase of both *I*_max_ and *Int*. 60 s values against nanoKAZ with each CTZ analog, respectively.

Interestingly, the wK/nK chimera could use CTZ as the preferred substrate and showed 8.6-fold higher activity than nanoKAZ (**Table 1A**). By contrast, the luminescence activity of the nK/wK chimera with CTZ was decreased significantly (**Table 1A**). Furthermore, the single reverse mutations of wK/nK at the nanoKAZ region (83–169 aa) with V90I (wK/nK-90I), E115P (wK/nK-115P), K124Q (wK/nK-124Q), I138Y (wK/nK-138Y), or R166N (wK/nK-166N) (**Fig 2B**) also failed to stimulate the luminescence activity with CTZ (**Table 1B**). Unexpectedly, wK/nK-124Q could only use *6h*-CTZ efficiently, similar to CTZ. Thus, the amino-terminal region (1–82 aa) of wild KAZ might contribute to the stimulation of luminescence activity with CTZ. From this result, we focused on the amino-terminal region of wild KAZ (wK: 1–82 aa) and produced three reverse mutants of nK-AQQL (E4A, R11Q, L18Q, and V27L), nK-KVA (R43K, I44V, and I54A), and nK-FLM (D68F, Q72L, and K75M) (**Fig 2C**). Among them, nK-AQQL showed the highest luminescence with 19.3-fold more activity than nanoKAZ with CTZ (**Table 1C**). Thus, the amino-terminal region of wild KAZ (1–27 aa) might directly or indirectly affect its substrate recognition for CTZ.

### Reverse mutant of QL-nanoKAZ shows high selective luminescence activity with CTZ

To elucidate the specific amino residue for luminescence stimulation with CTZ among four amino acid residues (E4A, R11Q, L18Q, and V27L) in nK-AQQL, four single-reverse mutants of nK-4A, nK-11Q, nK-18Q, and nK-27L were prepared and characterized (**Fig 2D**). The luminescence activities of nK-18Q and nK-27L mutants were stimulated 2.9-fold and 4.7-fold against nanoKAZ, respectively (**Table 2D**). Next, six double-reverse mutants of nK-4A11Q, nK-4A18Q, nK-4A27L, nK-11Q18L, nK-11Q27L, and nK-18Q27L were prepared (**Fig 2E**). The reverse mutant of nK-18Q27L (hereafter referred to as “QL-nanoKAZ”) showed the highest luminescence activity with CTZ. The reverse mutations at L18Q and V27L synergistically stimulated luminescence with CTZ. In addition, the reverse mutants of nK-27L, nK-11Q27L, and nK-18Q27L displayed luminescence with *6h*-CTZ, similar to nK-AQQL and wK/nK-124Q (**Table 2E**), though the reason for the high luminescence activity with *6h-*CTZ was unclear. A schematic representation of the relationships between the reverse mutants and their luminescence activities with CTZ and *bis-*CTZ are summarized in **Fig 2**, based on the results shown in **Tables 1** and **2**.

**Table 2:** Luminescence activities of reverse mutants of nanoKAZ using coelenterazine (CTZ) and CTZ analogs as a substrate.

nanoKAZ mutant ^a^ (ZZ domain fused protein)	Relative luminescence activity ^b^, *I*_max_ (*Int*. 60 ^c^)									
	CTZ		*h*-CTZ		*6h*-CTZ		*bis*-CTZ		*f*-CTZ	
***D***. nanoKAZ (nK)	1.0 ^d^	(1.0) ^e^	7.8	(3.8)	0.9	(0.8)	6.3	(5.3)	8.3	(4.2)
nK-4A	1.0	(1.0)	7.5	(3.3)	1.0	(0.9)	6.1	(4.9)	7.7	(3.6)
nK-11Q	1.3	(1.3)	7.2	(3.3)	1.1	(1.0)	6.6	(5.3)	7.4	(3.8)
nK-18Q	2.9	(2.5) ↑^f^	7.0	(3.1)	0.9	(1.2)	4.1	(4.3)	8.6	(4.3)
nK-27L	4.7	(3.9) ↑	7.9	(3.5)	3.3	(2.3) ↑	5.4	(4.4)	8.6	(4.3)
***E***. nK-4A11Q	1.2	(1.2)	7.4	(3.3)	0.9	(0.9)	7.1	(6.4)	7.6	(4.0)
nK-4A18Q	3.1	(2.5) ↑	8.7	(3.3)	1.0	(1.4)	4.5	(4.5)	9.1	(4.8)
nK-4A27L	4.6	(4.0) ↑	7.0	(3.1)	3.0	(1.9) ↑	5.5	(4.8)	8.6	(4.2)
nK-11Q18L	4.2	(3.3) ↑	8.7	(3.4)	1.6	(1.9) ↑	5.1	(5.0)	9.2	(4.9)
nK-11Q27L	5.6	(4.3) ↑	8.2	(3.4)	3.9	(3.1) ↑	5.4	(4.8)	9.3	(4.3)
nK-18Q27L	12.3	(11.9) ↑	7.9	(3.2)	3.0	(3.5) ↑	2.7	(3.1)	6.4	(3.4)

^a^ The mutated protein fused to ZZ domain was expressed in *E*. *coli* cells using a pCold-ZZ-P-X vector [24].

^b^ The luminescence activity (*n* = 2) was determined using an AB2200 luminometer with a 0.23% neutral density filter and shown as the relative intensity to that of nanoKAZ (nK) with CTZ.

^c^ Integration for 60 s in 0.1 s-intervals.

^d^ 1.8 × 10^5^ rlu/0.1 s.

^e^ 6.1 × 10^7^ rlu/60 s.

^f^ Vertical arrows (↑) indicate over 50% increase of both *I*_max_ and *Int*. 60 s values against nanoKAZ with each CTZ analog.

### Expression and purification of nanoKAZ, QL-nanoKAZ, and SNH-nanoKAZ from bacterial cells

To compare the luminescence properties of QL-nanoKAZ with those of nanoKAZ, SNH-nanoKAZ, and native OpLase, we expressed nanoKAZ, QL-nanoKAZ, and SNH-nanoKAZ as soluble proteins without the ZZ domain and highly purified them using Ni-chelate column chromatography (**Fig 3F**). It has been reported that SNH-nanoKAZ (teLuc) is a mutant of nanoKAZ with three amino acid substitutions (D19S, D85N, and C169H) and showed approximately 2-fold higher *I*_max_ value than FMZ using diphenylterazine (DTZ) (**Fig 1B**) with a red-shifted emission peak at 502 nm [15]. The protein concentrations of the purified luciferases were determined by amino acid composition analysis [27] and used for normalization of the luminescence activities and estimation of the luciferase concentration expressed in the mammalian cells.

**Fig 4:**
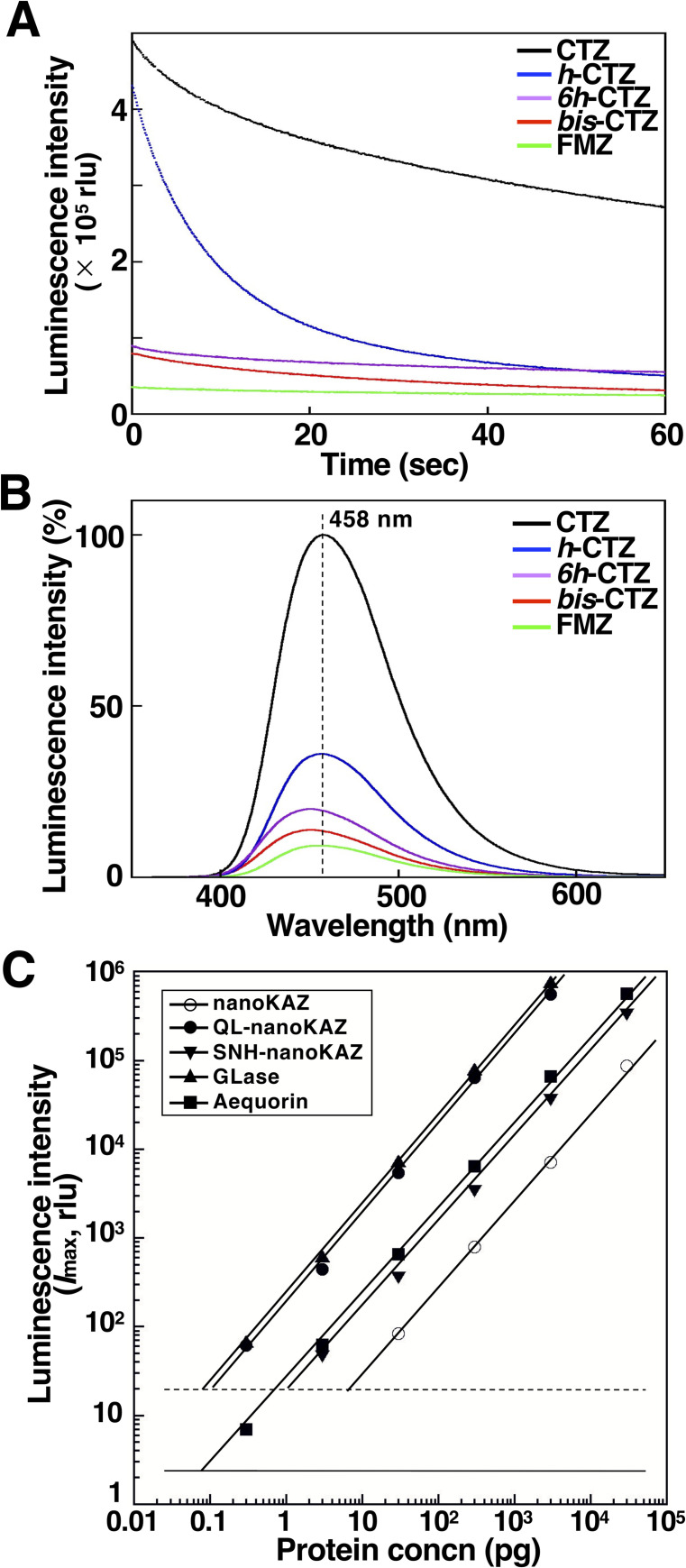
Luminescence properties of QL-nanoKAZ. A. Luminescence kinetics of QL-nanoKAZ with CTZ and its analogs as substrates. B. Normalized luminescence spectra of QL-nanoKAZ with CTZ and its analogs, based on the luminescence intensity of QL-nanoKAZ with CTZ. C. Linearity of luminescence intensity (*I*_max_) of QL-nanoKAZ with CTZ, in comparison with nanoKAZ, SNH-nanoKAZ, GLase, and aequorin at the protein concentrations of 0.3 pg to 3 ng (*n* = 6). Solid and dashed lines represent blank + 3 SD for aequorin and the CTZ-utilizing luciferases, respectively.

The luminescence kinetics and emission spectrum of QL-nanoKAZ were determined using CTZ and its four analogs. QL-nanoKAZ with CTZ showed the highest luminescence activity with slow decay kinetics (**Fig 4A**). The emission peaks of QL-nanoKAZ with CTZ and its analogs were around 458 nm, similar to native OpLase [4] and nanoKAZ [5] (**Fig 4B**). The linearity of luminescence activity for QL-nanoKAZ under various concentrations of CTZ was determined (**Fig 4C**) and the relative values of *I*_max_ and *Int*. 30 s were normalized using recombinant aequorin as a light standard (**Table 3**). Among the CTZ-utilizing luciferases, GLase is known to have the highest *I*_max_ value with fast decay kinetics [21,27]. The *I*_max_ value of QL-nanoKAZ with CTZ showed a similar order to that of GLase with more than 80-fold higher luminescence activity of nanoKAZ, and the luminescence kinetics was the slow decay pattern with a 2.6-fold higher value of *Int*. 30 s than that of GLase (**Table 3**). Thus, QL-nanoKAZ is a potential candidate for the reporter protein in the glow luminescence assay system.

**Table 3:** Comparison of luminescence activity of QL-nanoKAZ with other CTZ-utilizing luciferases using aequorin as a light standard.

Photoprotein or luciferases	Number of amino acids (Average mass value)	Relative luminescence intensity ^a^	
		*I*_max_	*Int*. 30 s
Aequorin	191 (21,632.20)	1.0 ^b^	1.0 ^c^
nanoKAZ (= nanoLuc)	191 (21,491.56)	0.12	3.4
QL-nanoKAZ	191 (21,520.55)	10.0	240
SNH-nanoKAZ (= teLuc)	191 (21,496.56)	0.53	15.0
GLase	174 (18,992.87)	12.3	92.0

^a^ Each purified recombinant luciferase and aequorin were dissolved in 0.1% bovine serum albumin (Sigma) in 50 mM Tris-HCl (pH 7.6)–10 mM EDTA. The luminescence reaction was initiated by adding 3 μL of each luciferase (300 pg) to 100 μL of PBS (Sigma) containing 1 μg of CTZ (1 μg/μL dissolved in ethanol). The luminescence activity (*n* = 6) was determined for 30 s using an AB2270 luminometer with an F2-cut filter. For aequorin assay, 100 μL of 50 mM CaCl_2_ in H_2_O was injected into 3 μL of aequorin (300 pg).

^b^ 2.1 × 10^7^ rlu/μg aequorin.

^c^ 2.0 × 10^8^ rlu/μg aequorin.

### Expression of QL-nanoKAZ in mammalian cells

OpLase is a secretory protein, but the catalytic component of wild KAZ could not be secreted from mammalian cells using its own signal peptide sequence [4,8] or other signal peptide sequences such that from GLase [8]. However, the signal peptide sequence for GLase (GLsp) has been successfully used for the secretion of nanoKAZ in CHO-K1 cells [5]. To evaluate QL-nanoKAZ as a reporter protein in mammalian cells, it was transiently expressed in CHO-K1 cells. The amount of QL-nanoKAZ secreted into the culture medium was similar to nanoKAZ and GLase (**Table 4**).

**Table 4:** Comparison of gene expression among nanoKAZ, QL-nanoKAZ, SNH-nanoKAZ, and GLase in CHO-K1 cells.

Luciferase: Expression vector	Relative luminescence intensity ^a^				Expressed proteins in cultured medium (μg/well, 24 h) ^d^
	Medium		Cell extracts		
	*I*_max_	*Int*. 10 s	*I*_max_	*Int*. 10 s	
nanoKAZ: pcDNA3-GLsp-dnKAZ	1.0 ^b^ (4.1)	1.0 ^c^ (3.7)	0.2 (8.4)	0.2 (7.0)	0.52
QL-nanoKAZ: pcDNA3-GLsp-QL-nK	31.3 (12.9)	30.5 (10.9)	5.3 (13.3)	5.5 (13.4)	0.52
SNH-nanoKAZ: pcDNA3-GLsp-SNH-nK	1.1 (4.3)	1.1 (0.9)	0.2 (2.0)	0.2 (1.9)	0.16
GLase: pcDNA3-GLsp-EpGLuc	71.7 (11.7)	56.5 (13.7)	17.6 (10.1)	16.7 (10.7)	0.20

^a^ CTZ used as a substrate. The luminescence activity was determined using an AB2270 luminometer with an F2-cut filter for 10 s in 0.1 s-intervals.

^b^ 1.4 × 10^6^ rlu/6-wells. The intra-assay coefficients of variations (CV%, *n* = 4) of individual assays are shown in parentheses.

^c^ 129.8 × 10^6^ rlu/6-wells. The intra-assay coefficients of variations (CV%, *n* = 4) of individual assays are shown in parentheses.

^d^ Estimated by the luminescence standard curve of each purified luciferase in Fig 4C.

As previously reported, nanoKAZ could be secreted into the culture medium even without a signal peptide sequence by the unknown mechanism [8]. Approximately 10% of nanoKAZ and QL-nanoKAZ without the signal peptide sequence was secreted into the culture medium [8], contrary to the report for nanoLuc by Hall *et al*. [9] (**Table 5**). Thus, QL-nanoKAZ with CTZ can be used as a reporter protein in the cytoplasm, similar to nanoKAZ/nanoLuc with *bis*-CTZ and *h-*CTZ [5]. However, based on the slow decay luminescence pattern of QL-nanoKAZ (**Fig 4A**), it was not suitable for real-time imaging of protein secretion from mammalian cells like GLase (unpublished results) [34,35].

**Table 5:** Expression of QL-nanoKAZ in the presence or absence of the secretory signal peptide sequence from *Gaussia* luciferase (GLsp) in CHO-K1 cells.

Expression -type	Expression vector	Relative luminescence intensity (*I*_max_, %) ^a^			
		CTZ		*bis*-CTZ	
		Medium	Cell extracts	Medium	Cell extracts
Secretion	pcDNA3-GLsp-dnKAZ	1.8	0.5	20.7	8.2
	pcDNA3-GLsp-QL-nK	100 ^b^	24.7	16.8	7.3
Cytoplasm	pcDNA3-dnKAZ	1.2	16.2	16.1	235
	pcDNA3-AQQL-nK	28.7	353	4.0	74.6
	pcDNA3-QL-nK	21.1	241	3.5	74.4

^a^ The luminescence activity (*n* = 2) was determined using an AB2270 luminometer with an F2-cut filter for 10 s in 0.1 s-intervals.

^b^ 17.6 × 10^6^ rlu/6-wells (*n* = 4).

### Luminescent products of CTZ catalyzed by CTZ-utilizing luciferases

**Fig 5:**
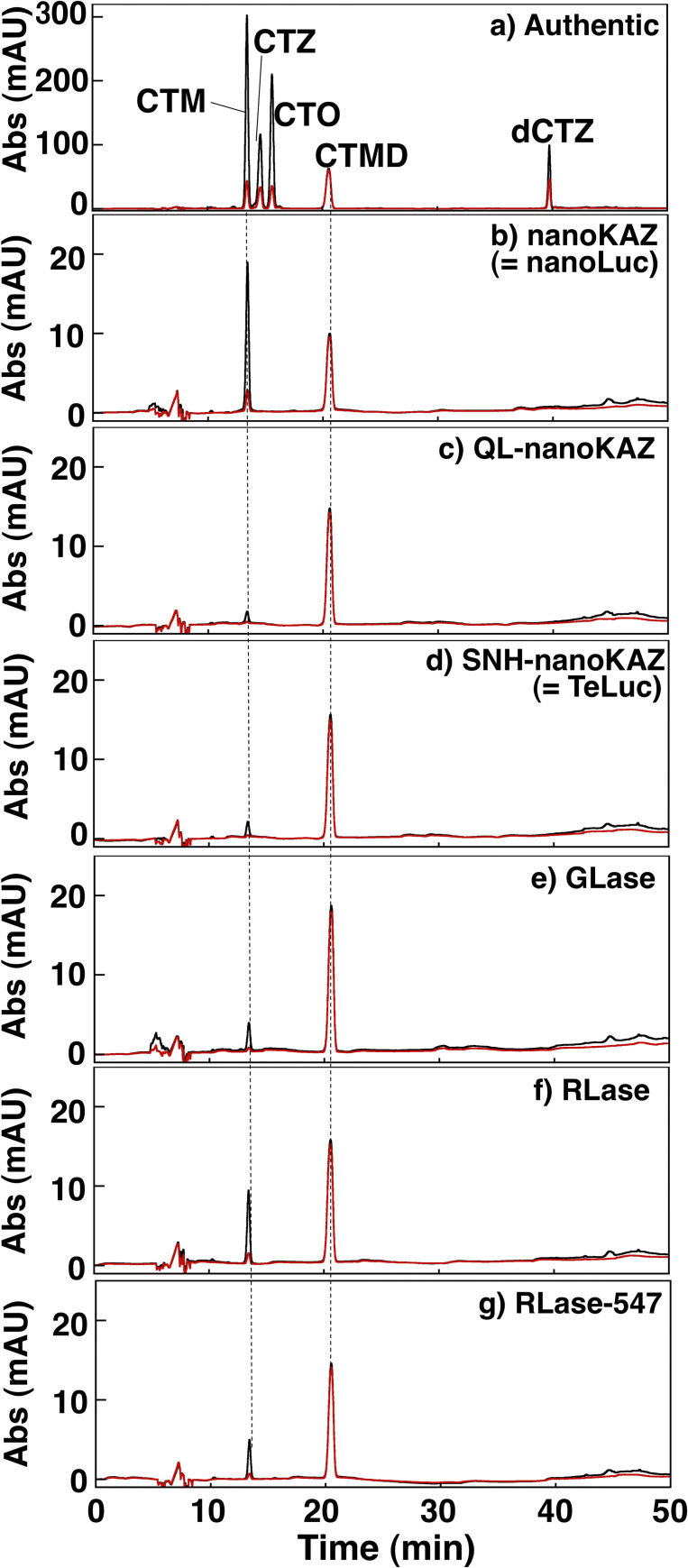
HPLC analyses of the reaction products from coelenterazine (CTZ) catalyzed by the CTZ-utilizing luciferases. Authentic samples (0.5 μg each) of coelenteramine (CTM), coelenterazine (CTZ), 3-benzyl-5-(4-hydroxyphenyl)pyrazin-2(1*H*)-one (CTO), coelenteramide (CTMD), and dehydrocoelenterazine (dCTZ). The black and red lines represent the absorbance at 280 and 330 nm, respectively. The amounts of the reaction products from CTZ by nanoKAZ, QL-nanoKAZ, SNH-nanoKAZ, GLase, RLase, and RLase-547 were determined using the predicted products as standard compounds (**Table 6**).

In the oxidation process of CTZ with O_2_ by luciferase, 2-peroxycoelenterazine (CTZ-OOH) might be an intermediate, from which CTMD is produced with light emission (**Fig 1A**). To identify the luminescent products of CTZ with the CTZ-utilizing luciferases, CTZ was incubated with each luciferase for 2 h and the products were analyzed by HPLC (**Fig 5**). The major and minor products from CTZ catalyzed by luciferases were CTMD and CTM, respectively. This was similar to the result obtained from CTZ-OOH in the Ca^2+^-triggered luminescence reaction of aequorin [36]. Recently, we investigated the degradation products from (*S*)-2-peroxycoelenterazine ((*S*)-CTZ-OOH) in aequorin under protein denaturing conditions and the major product was identified as CTM, but not CTMD, without light emission. In addition, we confirmed that CTMD was not hydrolyzed to CTM in aqueous solutions [36]. Thus, CTZ-OOH might be an intermediate in the luminescence reaction of CTZ catalyzed by these CTZ-utilizing luciferases.

**Table 6:** Reaction products of coelenteramine (CTM) and coelenteramide (CTMD) from coelenterazine (CTZ) by incubation of various CTZ-utilizing luciferases by HPLC analysis.

Luciferase + CTZ (2 μg, 473 pmol)	CTM ^a^ (pmol)	CTZ ^a^ (pmol)	CTMD ^a^ (pmol)	dCTZ ^a^ (pmol)	Products recovery from CTZ (%)	% of CTMD ^b^
nanoKAZ (= nanoLuc)	119	ND ^c^	201	ND	68	63
QL-nanoKAZ	8	ND	298	ND	65	97
SNH-nanoKAZ (= teLuc)	12	ND	321	ND	70	96
GLase	21	ND	371	ND	83	95
RLase	59	ND	325	ND	81	85
RLase-547	30	ND	276	ND	65	90
without luciferase ^d^	65	149	68	90	79	-

^a^ Estimated with the peak area on HPLC chart using authentic CTM, CTZ, CTMD, and dCTZ as standards.

^b^ Calculated with the equation of CTMD/(CTM + CTMD).

^c^ Not detected.

^d^ Data from ref. 19.

The efficiency of CTMD formation might be correlated with the luminescence activity of luciferases. Interestingly, even though CTZ is not a suitable substrate in nanoKAZ [5,9], CTZ was completely consumed and converted to CTMD and CTM with 63% and 37% in 2 h, respectively (**Table 6**). As we recently reported, the products after incubation of CTZ (473 pmol) in 50 mM Tris-HCl (pH 7.6) at 25°C for 2 h in the absence of luciferase were CTZ (149 pmol), CTMD (68 pmol), CTM (65 pmol), dCTZ (90 pmol) and an unknown product (**Table 6**) [19]. Because the formation of dCTZ was not detected in the reaction mixtures of CTZ with nanoKAZ and other luciferases, the CTM and CTMD formation from CTZ by nanoKAZ could be an enzymatic oxidation process. The low luminescence activity of nanoKAZ might be explained by that CTZ-OOH could not properly stabilized in the nanoKAZ molecule during the luminescence reaction and partially decomposes into CTM and CTMD without light emission [36].

### Substrate specificities of nanoKAZ, QL-nanoKAZ, SNH-nanoKAZ, and native OpLase for CTZ analogs

As previously reported, native OpLase showed broad substrate specificity [11], and the catalytic component of 19 kDa protein (wild KAZ) in OpLase also showed similar substrate specificity [4,13]. As summarized in **Table 7,** nanoKAZ and SNH-nanoKAZ showed lower luminescence activity with CTZ, but other profiles of substrate specificity were similar to those of OpLase and wild KAZ. By contrast, QL-nanoKAZ could use CTZ and *h*-CTZ as the preferred substrates, similar to OpLase [11] and wild KAZ [13]. The *I*_max_ value of QL-nanoKAZ with CTZ was 2.6- and 5.3-folds higher than that of nanoKAZ with *bis*-CTZ and FMZ, respectively (**Table 7**). Furthermore, QL-nanoKAZ with all C2-modified CTZ analogs possessing the hydroxy moiety (-OH) at the C6 benzyl group retained higher luminescence activity than those of nanoKAZ and SNH-nanoKAZ. Among these mutants, similar luminescence activities were observed for *n*-CTZ and *hcp*-CTZ, suggesting that the C2- and C8-groups of CTZ might not be essential for binding to these luciferases (**Table 7**). In QL-nanoKAZ, stabilization of the hydroxy moiety at the C6-benzyl group of CTZ with specific amino acid residue(s) in the protein might be crucial to stimulate the luminescence activity with CTZ. In addition, the amino acid residues that interact with the C3-carbonyl group of the imidazopyrazinone structure also might be required to stabilize CTZ analogs lacking the hydroxy moiety at the C6-benzyl group of CTZ (i.e., *bis*-CTZ, *6h*-CTZ, and FMZ).

**Table 7:** Substrate specificities and luminescence properties for purified QL-nanoKAZ, nanoKAZ, SNH-nanoKAZ, and native *Oplophorus* luciferase (OpLase).

Coelenterazine analogs		QL-nanoKAZ				nanoKAZ				SNH-nanoKAZ				OpLase	
Prefix	Substitution	*I*_max_ (%)	*Int*. 60 s (%)	*λ*_max_ (nm)	FWHM (nm)	*I*_max_ (%)	*Int*. 60 s (%)	*λ*_max_ (nm)	FWHM (nm)	*I*_max_ (%)	*Int*. 60 s (%)	*λ*_max_ (nm)	FWHM (nm)	*I*_max_ (%)	*Int*. 60 s (%)
**CTZ**	**None**	**100** ^**a**^	**100** ^**b**^	**458**	**74**	**3**	**4**	**457**	**74**	**11**	**14**	**460**	**75**	**100** ^**c**^	**100** ^***d***^
***bis***	**2: -CH**_**2**_ **C**_**6**_ **H**_**5**_ **6: -C**_**6**_ **H**_**5**_	**16**	**14**	**451**	**74**	**38**	**48**	**451**	**74**	**38**	**40**	**454**	**75**	**38**	**44**
***h***	**2: -CH**_**2**_ **C**_**6**_ **H**_**5**_	**86**	**36**	**457**	**72**	**64**	**49**	**458**	**72**	**40**	**25**	**460**	**77**	**73**	**44**
***6h***	**6: -C**_**6**_ **H**_**5**_	**18**	**20**	**451**	**75**	**2**	**3**	**454**	**75**	**8**	**9**	**454**	**75**	**4**	**4**
**FMZ**	**2: 2-furanylmethyl** **6: -C**_**6**_ **H**_**5**_	**7**	**9**	**451**	**73**	**19**	**26**	**451**	**70**	**17**	**23**	**454**	**73**	**16**	**18**
***hcp***	**2: -CH**_**2**_ **C**_**6**_ **H**_**5**_ **8: -CH**_**2**_ **C**_**5**_ **H**_**9**_ **(*c*)**	**26**	**14**	**446**	**70**	**10**	**8**	**449**	**70**	**19**	**12**	**447**	**70**	**24**	**19**
***ameh***	**2: 1-phenylethyl**	**10**	**6**	**453**	**74**	**1**	**1**	**464**	**77**	**3**	**3**	**460**	**75**	**3**	**3**
***f***	**2: -CH**_**2**_ **C**_**6**_ **H**_**4**_ **F(*p*)**	**64**	**21**	**453**	**70**	**71**	**52**	**453**	**70**	**45**	**30**	**454**	**69**	**39**	**37**
***3iso***	**2: -CH**_**2**_ **C**_**6**_ **H**_**4**_ **OH(*m*)**	**53**	**40**	**457**	**73**	**6**	**7**	**460**	**74**	**14**	**17**	**459**	**73**	**23**	**24**
***meo***	**2: -CH**_**2**_ **C**_**6**_ **H**_**4**_ **OCH**_**3**_ **(*p*)**	**53**	**25**	**455**	**72**	**31**	**26**	**456**	**73**	**23**	**19**	**458**	**73**	**32**	**27**
***3meo***	**2: -CH**_**2**_ **C**_**6**_ **H**_**4**_ **OCH**_**3**_ **(*m*)**	**84**	**34**	**457**	**72**	**45**	**37**	**457**	**71**	**37**	**28**	**460**	**72**	**26**	**26**
***cf3***	**2: -CH**_**2**_ **C**_**6**_ **H**_**4**_ **CF**_**3**_ **(*p*)**	**37**	**17**	**454**	**73**	**19**	**11**	**454**	**71**	**20**	**14**	**455**	**73**	**7**	**7**
***i***	**2: -CH**_**2**_ **C**_**6**_ **H**_**4**_ **I(*p*)**	**24**	**11**	**453**	**72**	**11**	**6**	**460**	**75**	**11**	**7**	**457**	**74**	**7**	**6**
***me***	**2: -CH**_**2**_ **C**_**6**_ **H**_**4**_ **CH**_**3**_ **(*p*)**	**65**	**26**	**457**	**74**	**24**	**18**	**458**	**75**	**21**	**15**	**461**	**73**	**20**	**17**
***et***	**2: -CH**_**2**_ **C**_**6**_ **H**_**4**_ **CH**_**2**_ **CH**_**3**_ **(*p*)**	**40**	**14**	**457**	**72**	**18**	**10**	**457**	**73**	**15**	**11**	**460**	**74**	**13**	**9**
***3me***	**2: -CH**_**2**_ **C**_**6**_ **H**_**4**_ **CH**_**3**_ **(*m*)**	**49**	**21**	**455**	**73**	**44**	**35**	**457**	**71**	**26**	**21**	**460**	**72**	**48**	**38**
***n***	**2: 2-naphthylmethyl**	**17**	**12**	**451**	**70**	**13**	**7**	**453**	**71**	**12**	**9**	**455**	**72**	**11**	**6**

The assay conditions are as follows: The luminescence reaction was initiated by adding 1 μg of CTZ analogs (dissolved in ethanol) to 100 μL of 30 mM Tris-HCl (pH 7.6)–10 mM EDTA containing 3 μL of 15 ng protein (QL-nanoKAZ, nanoKAZ, or SNH-nanoKAZ) and 270 ng of OpLase. The luminescence activity (*n* = 3) was determined using an AB2270 luminometer with an F2-cut filter.

^a^ 4.9 × 10^5^ rlu/0.1 s.

^b^ 2.0 × 10^8^ rlu/60 s.

^c^ 1.8 × 10^5^ rlu/0.1 s.

^d^ 8.6 × 10^7^ rlu/60 s.

### Effect of deaza-analogs for CTZ and CTZ-OOH on luciferase activity

The chiral deaza-analogs of (*S*)- and (*R*)-deaza-CTZ ((*S*/*R*)-daCTZ) for CTZ and (*S*)-2- and (*R*)-2-hydroxymethyl-deaza-CTZ ((*S*/*R*)-HM-daCTZ) for CTZ-OOH (**Fig 1C**) have been used as inhibitors to investigate the oxidation process of CTZ with O_2_ in RLase [12] and the regeneration process of the calcium-binding photoprotein, aequorin, from CTZ and O_2_ [19]. The inhibitory effect on luminescence activity of various CTZ-utilizing luciferases with CTZ was examined in the presence of each deaza-analog (**Table 8**). As previously reported, RLase was inhibited by (*S*/*R*)-daCTZ and by (*S*)-HM-daCTZ [12]. We proposed that the binding of CTZ to RLase might be a non-stereospecific process, while the following oxidation of CTZ with O_2_ to CTZ-OOH might be a stereospecific process. Interestingly, the luminescence activities of GLase and RLase-547 were clearly inhibited by (*S*)-HM-daCTZ and (*R*)-HM-daCTZ, respectively, suggesting that the addition of O_2_ to CTZ might be a stereospecific process. Notably, the inhibition stereospecificity of RLase-547 by (*R*) form of HM-daCTZ was different from that of RLase by (*S*) form of HM-daCTZ. This result suggested that the addition of O_2_ to CTZ might be proceeded in the opposite direction between RLase and RLase-547 (**Table 8**).

**Table 8:** Inhibition of luminescence activity of CTZ-utilizing luciferases with deaza-coelenterazine (daCTZ) analogs as inhibitors.

Inhibitors	Relative luminescence activity (*I*_max_, %)							
	OpLase ^a^	nanoKAZ ^a^	QL-nanoKAZ ^a^	SNH-nanoKAZ ^a^	GLase ^a^	RLase ^a^	RLase-547 ^a^	Aequorin ^c^
None	100 ^b^	100 ^b^	100 ^b^	100 ^b^	100 ^b^	100 ^b^	100 ^b^	100
(*S*)-daCTZ	2.1	26.8	0.4	21.1	0.4	0.1	0.04	5.8
(*R*)-daCTZ	5.1	16.6	0.4	12.7	0.5	0.2	0.1	33.4
(*S*)-HM-daCTZ	19.7	62.8	13.1	55.2	5.3	25.2	3.1	8.7
(*R*)-HM-daCTZ	33.5	60.5	18.8	54.6	23.5	1.4	27.0	92.1

^a^ The reaction mixture contained each luciferase in 200 μL of 30 mM Tris-HCl–10 mM EDTA and was incubated with each inhibitor (1 μg/1 μL dissolved in ethanol: (*S*/*R*)-daCTZ, 1.2 × 10^−4^ M; (*S*/*R*)-HM-daCTZ; 1.2 × 10^−4^ M) for 1 min. Then, the luminescence reaction was initiated by mixing with CTZ (1 μg/1 μL dissolved in ethanol: 1.2 × 10^−4^ M) and the luminescence activity (*n* = 3) was determined using an AB2270 luminometer with an F2-cut filter. The luciferase concentrations used for assay were as follows: OpLase, 90 ng (4.2 × 10^−9^ M); nanoKAZ, 20 ng (4.7 × 10^−9^ M); QL-nanoKAZ, 5 ng (1.2 × 10^−9^ M); SHN-nanoKAZ, 15 ng (3.5 × 10^−9^ M); GLase, 5 ng (1.3 × 10^−9^ M); RLase, 15 ng (2.0 × 10^−9^ M); RLase, 15 ng (2.0 × 10^−9^ M).

^b^ The *I*_max_ value without inhibitors is as follows: OpLase, 3.7 × 10^4^ rlu; nanoKAZ, 3.6 × 10^4^ rlu; QL-nanoKAZ, 8.9 × 10^5^ rlu; SNH-nanoKAZ, 1.3 × 10^5^ rlu; GLase, 8.8 × 10^5^ rlu; RLase, 5.9 × 10^5^ rlu; RLase-547, 1.2 × 10^5^ rlu.

^c^ Data obtained from ref. 19.

Under the same assay conditions, the luminescence activity of QL-nanoKAZ was significantly inhibited by (*S*/*R*)-daCTZ, similar to GLase, RLase, and RLase-547. However, nanoKAZ and SNH-nanoKAZ were moderately inhibited by (*S*/*R*)-daCTZ. Thus, the binding affinity of CTZ to QL-nanoKAZ might be comparable to that of GLase, RLase, and RLase-547, showing high luminescence activity.

On the other hand, the luminescence activity of OpLase, nanoKAZ, QL-nanoKAZ, and SNH-nanoKAZ were not selectively inhibited by (*S*)- or (*R*)-HM-daCTZ, suggesting that the oxidation step of CTZ with O_2_ by these luciferases might be a non-stereospecific process. However, it is possible that (*S/R*)-HM-daCTZ binds less efficiency to the catalytic pocket of luciferase due to steric hindrance by the hydroxymethyl group of daCTZ.

### Crystal structure of QL-nanoKAZ and substrate specificity for CTZ

Based on the results showing inhibitory effect of deaza-CTZ analogs against QL-nanoKAZ (**Table 8**), we attempted to determine the complex structure of daCTZ or HM-daCTZ with QL-nanoKAZ, but were unsuccessful. However, in the absence of deaza-CTZ analogs, the structure of QL-nanoKAZ was determined at 1.70 Å resolution (PDB ID: 7VSX). The statistical values of data collection and structure refinement are summarized in **Table 9**.

**Table 9:** Statistics of data collection and structure refinement.

*Data collection and processing*	
Beamline	BL26B2
Space group	*I*222
Unit-cell parameter	
*a*, *b*, *c* (Å)	60.8, 76.0, 103.8
*α*, *β*, *γ* (Å)	90.0, 90.0, 90.0
Wavelength (Å)	1.000
Resolution range (Å)	50–1.70 (1.80–1.70)
Redundancy	7.3 (7.2)
Completeness (%)^a^	98.5 (99.7)
*R*_sym_^b^ (%)^a^	7.0 (88.7)
*I/σ(I)*^a^	15.1(2.2)
No. monomers/asymmetric unit	1
***Model refinement***	
No. of reflections	26752
No. of protein atoms	1355
No. of water molecules	174
*R*_work_/*R*_free_^c^ (%)	18.2/20.7
r.m.s.d. for bond length (Å)	0.014
r.m.s.d. for bond angles (˚)	1.2
***Residues in the Ramachandran plot***	
Favored region (%)	95.9
Allowed regions (%)	4.1
PDB entry	7VSX

^a^ Statistics for the highest resolution shell are given in parentheses.

^b^ *R*_sym_ = (∑_*h*_∑_*i*_|*I*_*hi*_–‹*I*_*h*_›|/∑_*h*_∑_*i*_|*I*_*hi*_|) where *h* indicates unique reflection indices and *i* indicates symmetry equivalent indices.

^c^ *R*_work_ = ∑|*F*_obs_–*F*_calc_|/∑*F*_obs_ for all reflections and *R*_free_ was calculated using randomly selected reflections (6%).

**Fig 6:**
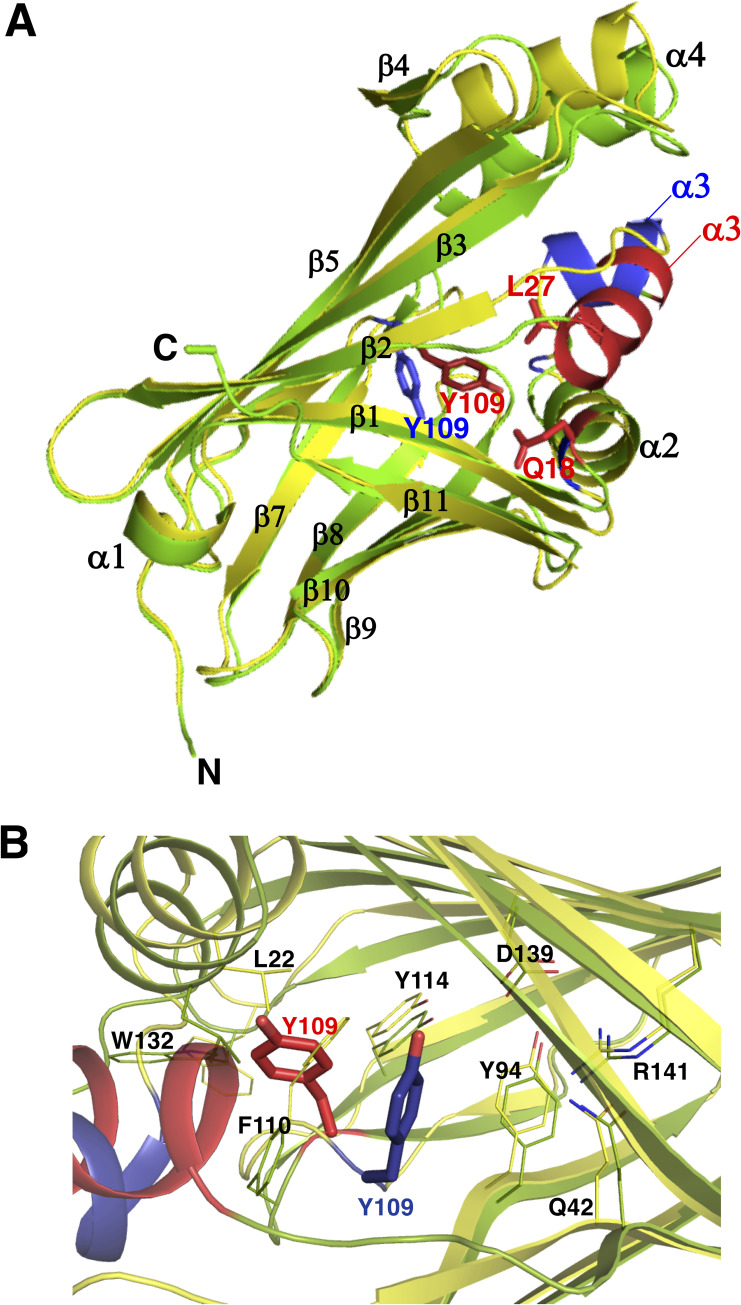
Crystal structure of QL-nanoKAZ. A. Comparison of the crystal structures between nanoKAZ and QL-nanoKAZ. A superimposed structure of QL-nanoKAZ (green, PDB ID: 7VSX) on that of nanoKAZ (yellow, PDB ID: 5B0U). Red color in QL-nanoKAZ and blue color in nanoKAZ indicate the differences in structure at the α3-helices and Tyr 109, respectively. B. The amino acid residues around Tyr 109 in QL-nanoKAZ and nanoKAZ. A cartoon representation of QL-nanoKAZ (green) superimposed on nanoKAZ (yellow) around Tyr 109. Red- and blue colors at the α3-helices and Tyr 109 are from QL-nanoKAZ and nanoKAZ, respectively.

**Fig 7:**
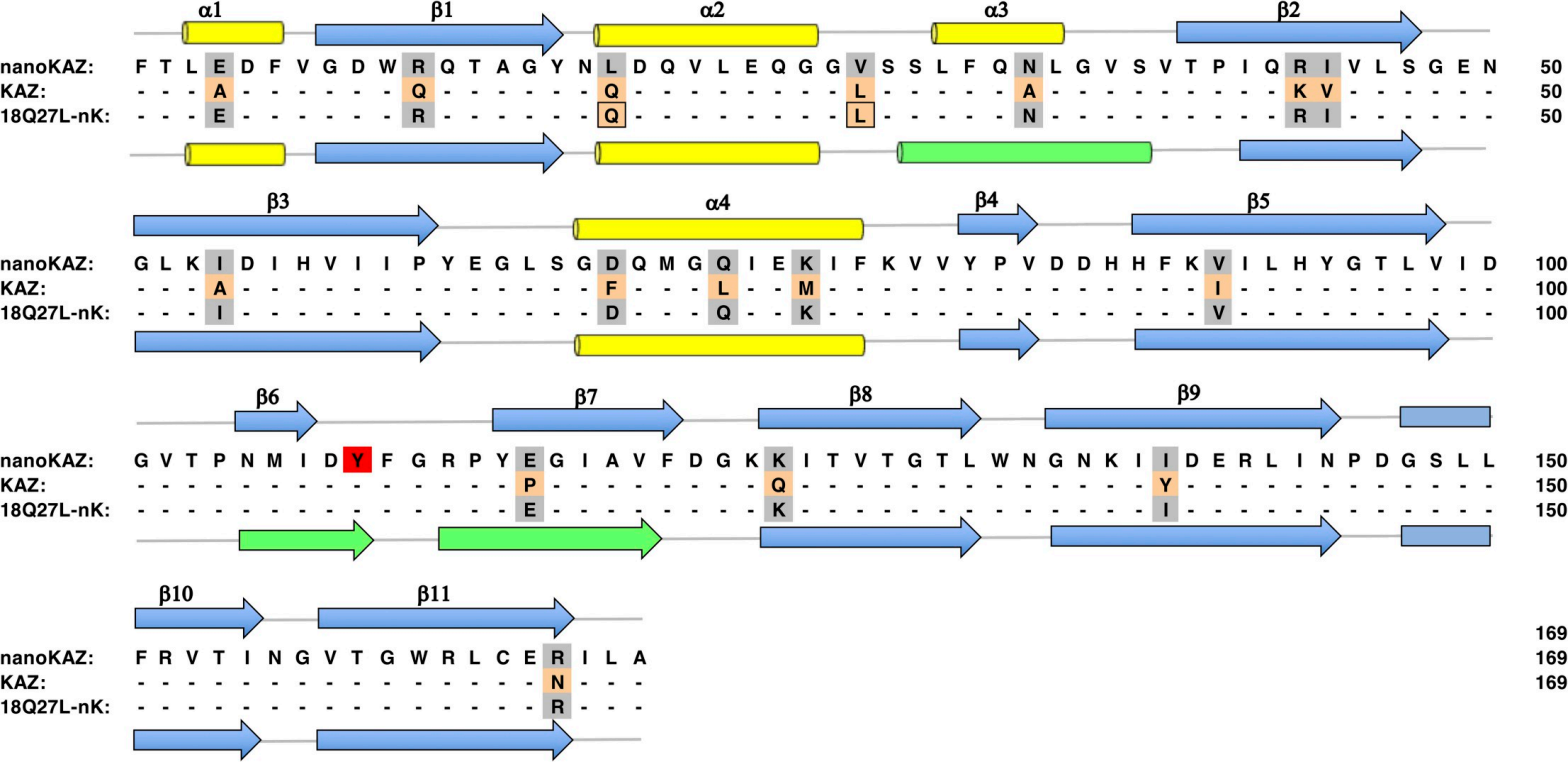
Comparison of the secondary structures between nanoKAZ and QL-nanoKAZ. The amino acid sequences of nanoKAZ and QL-nanoKAZ are shown with their positions of the secondary structure, and the letters highlighted in orange indicate the substituted 16 amino acid residues in wild KAZ to prepare reverse mutations of nanoKAZ. The cylinders and arrows indicate the regions of α-helices (yellow, α1–α4) and β-strands (blue, β1–β11), respectively. The green in the cylinder (α3) and the arrows (β6 and β7) in QK-nanoKAZ indicate the structural differences compared to nanoKAZ. Tyr 109 is highlighted in red.

The overall structure of QL-nanoKAZ substituted at L18Q and V27L was almost the same as that of nanoKAZ (PDB ID: 5B0U) (**Fig 6A**). But some structural differences were observed at the α3-helix (29–37 aa), β6-sheet (105–109 aa), and β7-sheet (112–119 aa) (**Fig 7**). Interestingly, the amino acid residue at Tyr 109 in the β6-sheet showed significant change in position, which might be hydrogen bond formation between the hydroxy group of Tyr 109 and the C6-hydroxy group of CTZ, increasing the affinity with CTZ (**Fig 6B**).

Recently, we determined the structures of a complex of apoAequorin (apoprotein of aequorin) with (*S*)-daCTZ (PDB ID: 7EG2) or (*S*)-HM-daCTZ (PDB ID: 7EG3). The stabilization mechanisms of daCTZ and HM-daCTZ in apoAequorin were identical to that of the complex of apoAequorin and (*S*)-CTZ-OOH (aequorin: PDB ID: 1EJ3) [19]. Thus, the C6-hydroxy and the C3-carbonyl groups in both CTZ and CTZ-OOH were stabilized by the hydrogen-bonding interactions *via* three amino acid residues (His 16, Tyr 82, and Trp 86) and His 169, respectively. On the other hand, the Ca^2+^-triggered *Renilla* luciferin-binding protein (RLBP) is a complex of apoRLBP (apoprotein of RLBP) and CTZ [37,38], and apoRLBP can also bind *h-*CTZ and *bis*-CTZ [38]. The crystal structure of RLBP from *R*. *muelleri* containing CTZ has been determined (PDB ID: 2HPS) [39] and CTZ was stabilized with hydrogen-bonding interactions at the C2-hydroxy group with both Tyr 36 and Arg 19, the C6*-*hydroxy group with Asp 183 and Lys 139 though two H_2_O molecules, the C3-carbonyl group with Arg 22, and the N(7) nitrogen with Phe 190. As apoRLBP could stably bind to both *h*-CTZ and *bis-*CTZ to form semi-synthetic RLBP [38], the C3-carbonyl group and the N(7) nitrogen in CTZ might be responsible for the stabilization with apoRLBP without interacting the C2- and C6-hydroxy groups of CTZ. Thus, there are variations in the stabilization pattern of CTZ by the amino acid residues in each protein molecule.

In QL-nanoKAZ, CTZ was the preferred substrate over *bis*-CTZ and FMZ, which lack the C2- and C6-hydroxy groups of CTZ. When the C2-CTZ analogs were used as a substrate, the decrease in luminescence activity of QL-nanoKAZ was not significant compared to CTZ (**Table 6**). Thus, the C2*-*hydroxy group of CTZ might not be essential for binding QL-nanoKAZ, similar to nanoKAZ and SNH-nanoKAZ. Furthermore, *hcp-*CTZ with a C8 substitution in CTZ could be efficiently used as a substrate by QL-nanoKAZ, nanoKAZ, and SNH-nanoKAZ, indicating that the C8-phenyl group of CTZ was also not required for molecular recognition of CTZ by QL-nanoKAZ. From these results, the high luminescence activity of QL-nanoKAZ with CTZ might be explained by the high affinity between the C6-hydroxy group of CTZ and QL-nanoKAZ. The reverse mutations at V18Q and V27L in nanoKAZ caused local changes in its structure, resulting in stabilization of CTZ for efficient oxidation with O_2_. Although the binding cavity in nanoKAZ for CTZ was not determined, Tyr 109 seems to be the potential amino acid residue that stabilizes the C6*-*hydroxy group of CTZ with hydrogen-bonding interactions, resulting in efficient oxidation of CTZ.

## Conclusions

To investigate the differences in substrate specificities between wild KAZ and nanoKAZ toward CTZ and *bis*-CTZ, the reverse nanoKAZ mutants substituted with the identical amino acid residue of wild KAZ at the same position were prepared. Among these mutants, a reverse mutant substituted with L18Q and V27L (QL-nanoKAZ) showed the highest luminescence activity with CTZ, and the luminescence properties of QL-nanoKAZ were compared with those of the CTZ-utilizing luciferases including nanoKAZ, *Renilla* luciferase, and *Gaussia* luciferase. The results showed that QL-nanoKAZ is an ideal candidate for the reporter protein in various luminescence assay systems. Furthermore, the crystal structure of QL-nanoKAZ was determined, showing that the reverse mutations at V18Q and V27L in nanoKAZ causes local structural changes and might enhance the binding affinity to CTZ and lead to efficient oxidized of CTZ with O_2_ to emit light.

## Supporting information

S1 Fig Photograph of QL-nanoKAZ crystal for structural analysis. (DOC)

S1 Table Primer list used for site-directed mutagenesis to prepare reverse mutant genes for nanoKAZ by PCR. (DOC)

S2 Table Purification of QL-nanoKAZ from 800 mL of cultured *E*. *coli* cells using a Ni-chelate column. (DOC)

S1 Raw images (PDF)

## Abbreviations

OpLase: *Oplophorus* luciferase
KAZ: the catalytic 19 kDa protein of OpLase
nanoKAZ (= nanoLuc): a KAZ mutant with 16-amino acid substitutions;QL-nanoKAZ, a nanoKAZ mutant reversed to L18Q and V27L
SNH-nanoKAZ (= teLuc): a mutant of nanoKAZ with D19S, D85N, C169H
RLase: *Renilla* luciferase
RLase-547 (= RLuc8.6–547): *Renilla* luciferase mutant with an emission peak at 547 nm
GLase: *Gaussia* luciferase
GLsp: the signal peptide sequence of GLase
RLBP: *Renilla* luciferin-binding protein
apoAequorin: apoprotein of aequorin
CTZ: coelenterazine
CTZ-OOH: 2-peroxycoelenterazine
CTMD: coelenteramide
CTM: coelenteramine
CTO: 3-benzyl-5-(4-hydroxyphenyl)pyrazin-2(1*H*)-one
dCTZ: dehydrocoelenterazine
FMZ: furimazine
daCTZ: deaza-coelenterazine
I_max_: maximum intensity of luminescence
Int: integrated luminescence intensity in 0.1 s-intervals
*λ*_max_: maximum wavelength of luminescence
FWHM: a full width of half maximum rlu, relative light units
CHO-K1 cell: Chinese hamster ovary-K1 cell
aa: amino acid residues
