# Supplementary figures and images for: Reverse mutants of the catalytic 19 kDa mutant protein (nanoKAZ/nanoLuc) from Oplophorus luciferase with coelenterazine as preferred substrate
Source: PLoS One. 2022 Sep 21;17(9):e0272992. doi: 10.1371/journal.pone.0272992 (PMC9491549; doi:10.1371/journal.pone.0272992)

**S1 Fig.**

**Photograph of QL-nanoKAZ crystal for structural analysis**


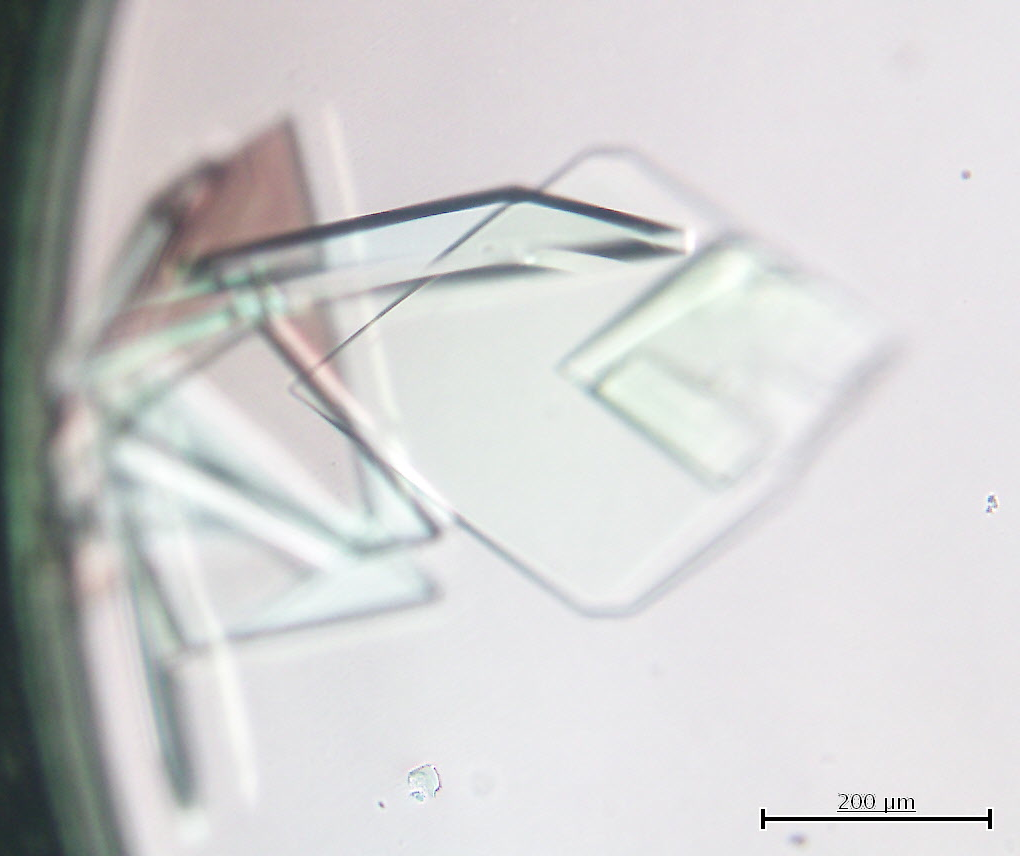

Supplement: S1 Fig — (DOC) [file pone.0272992.s001.doc]

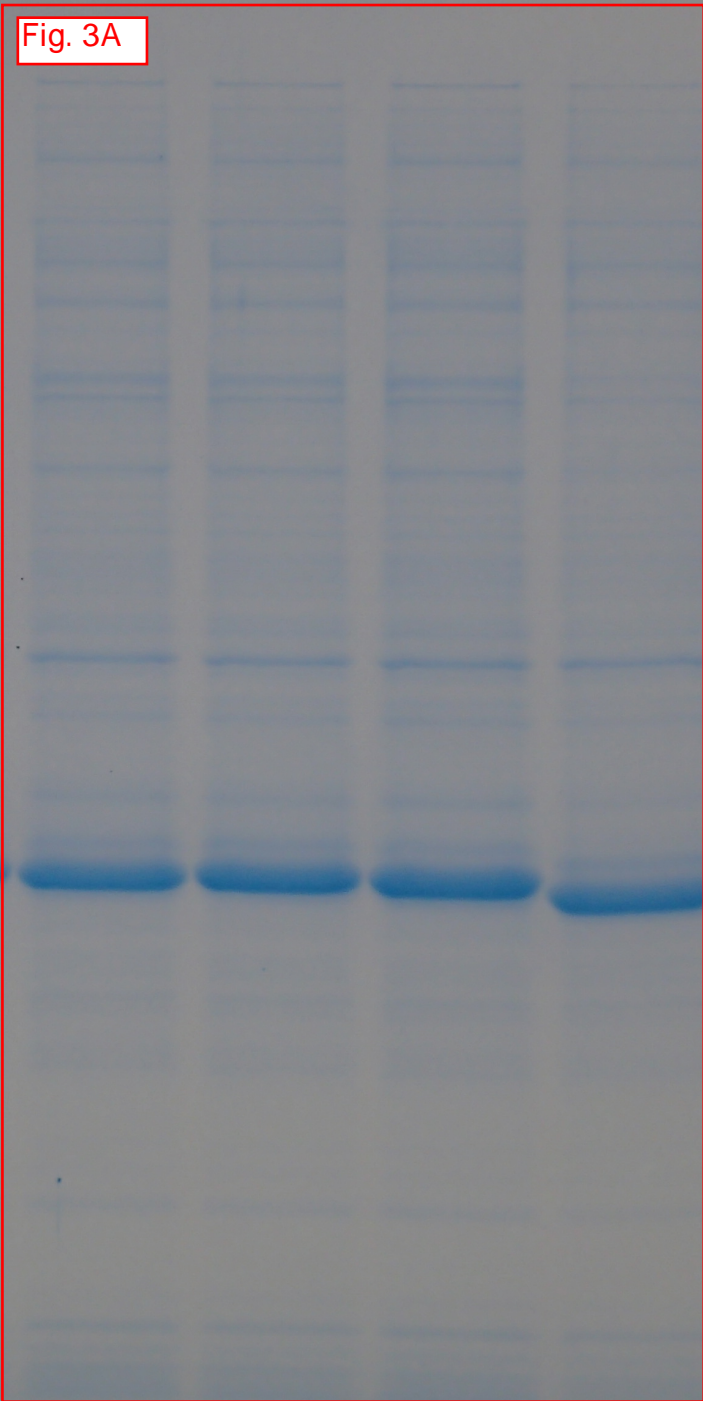

Fig. 3B

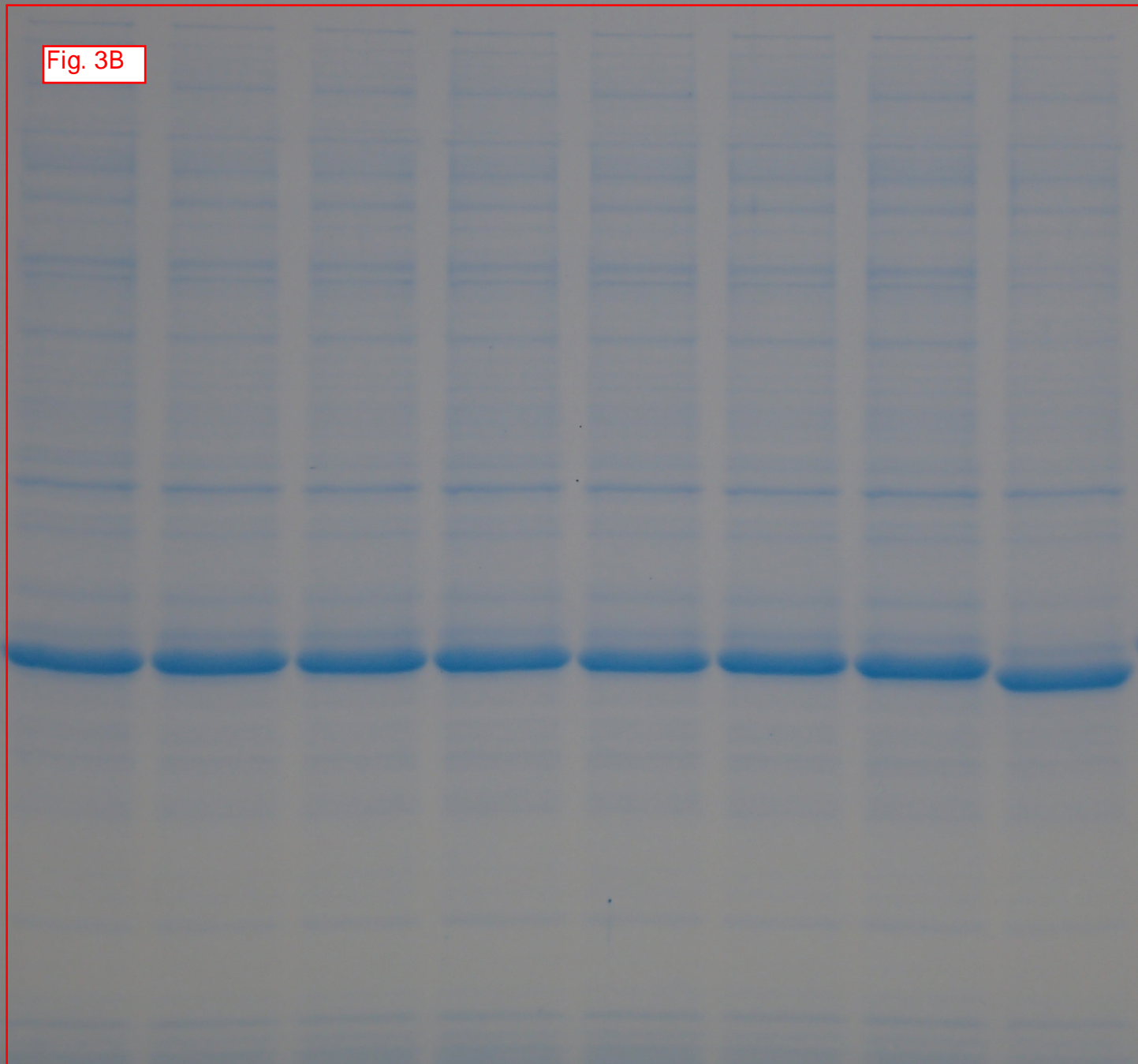

Fig. 3C

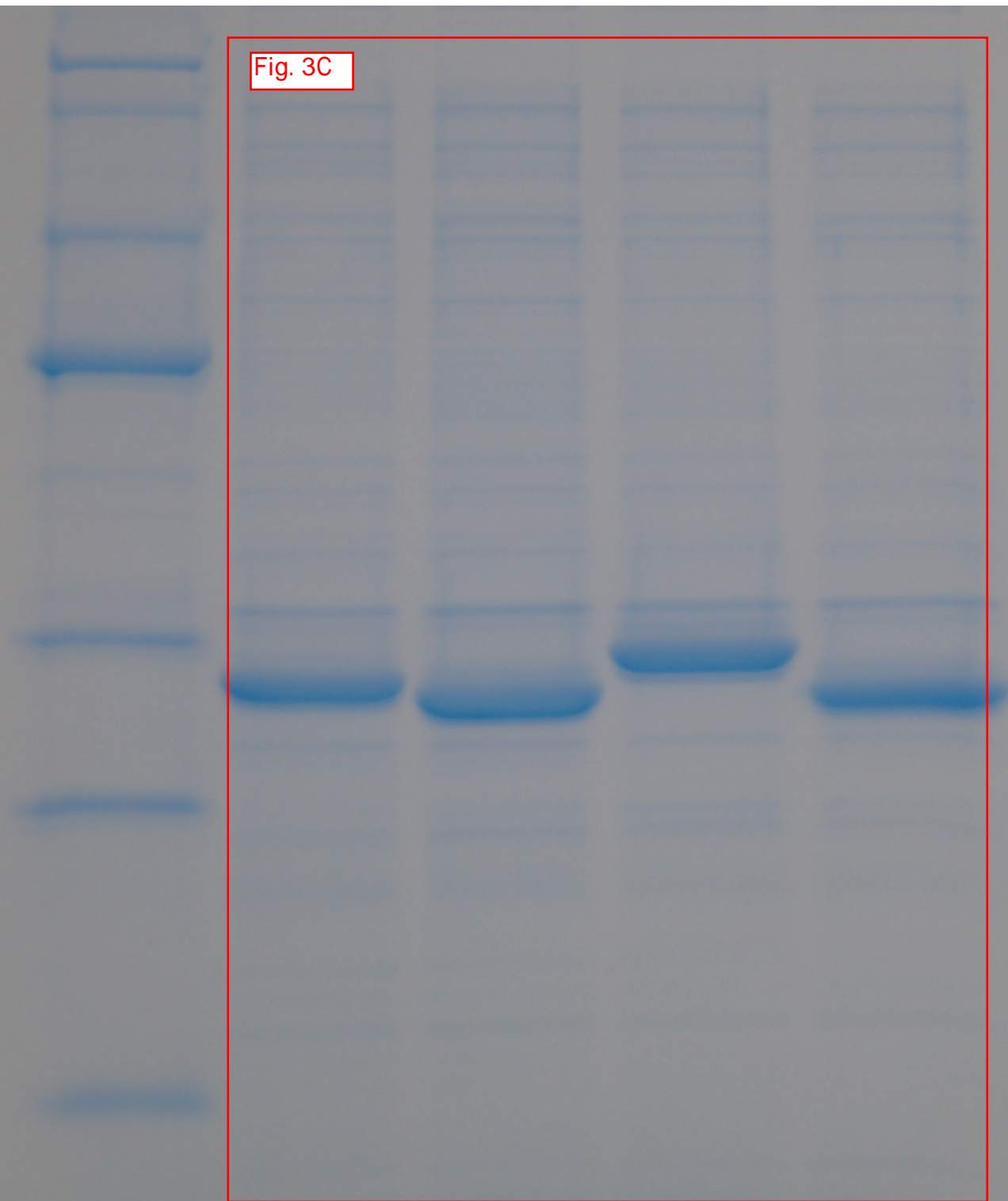

Fig. 3D

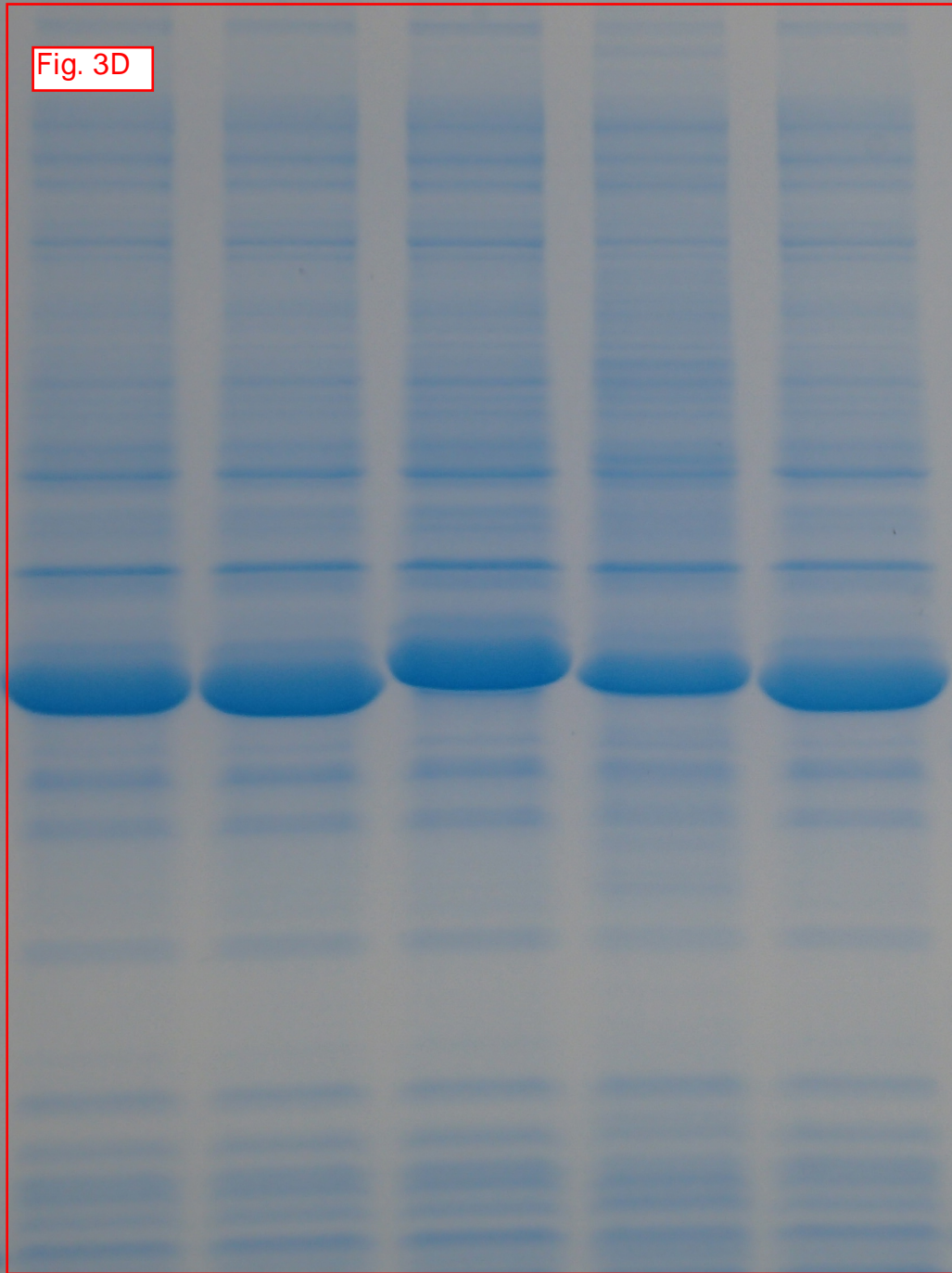

Fig. 3E

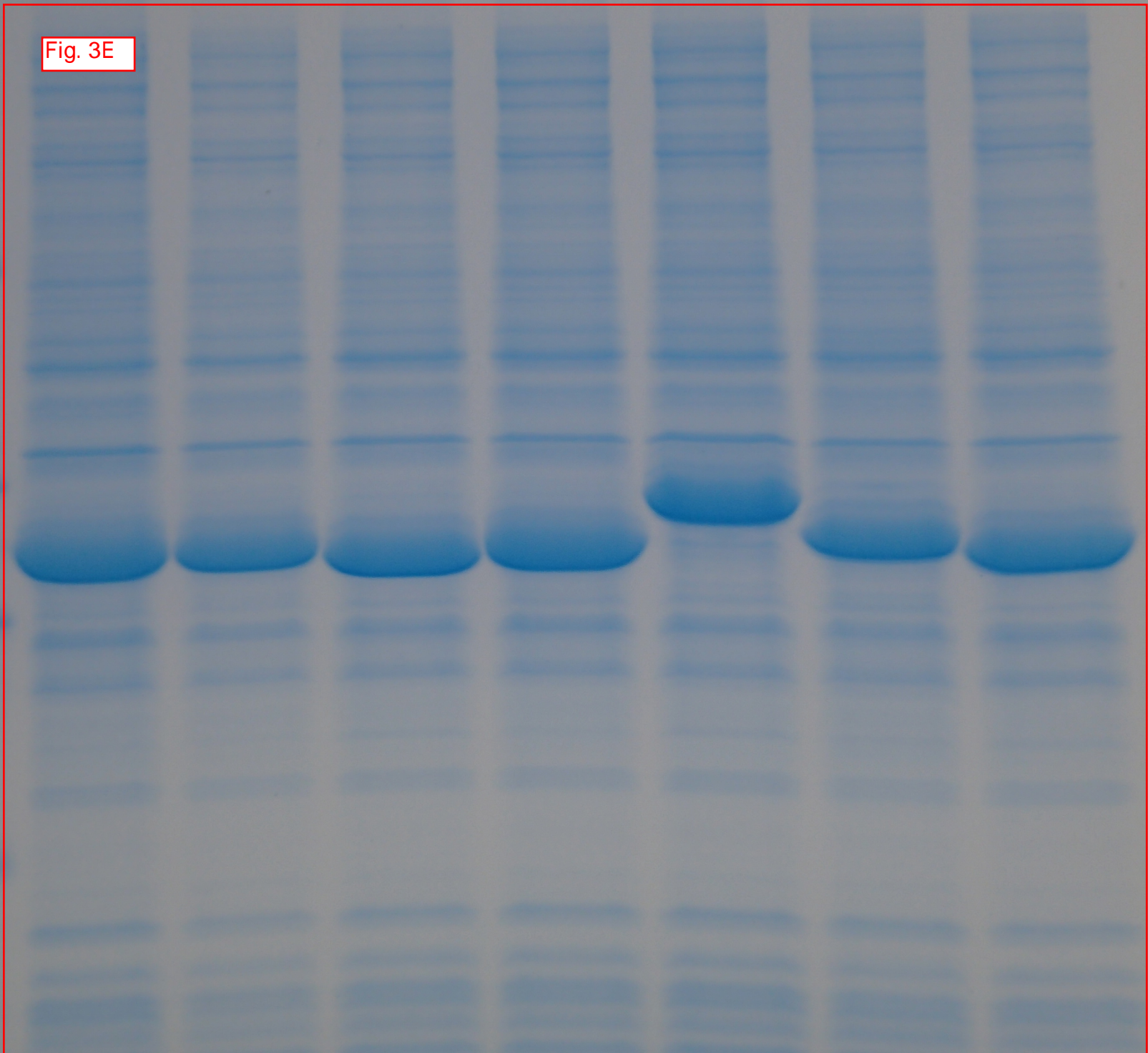

Fig. 3F

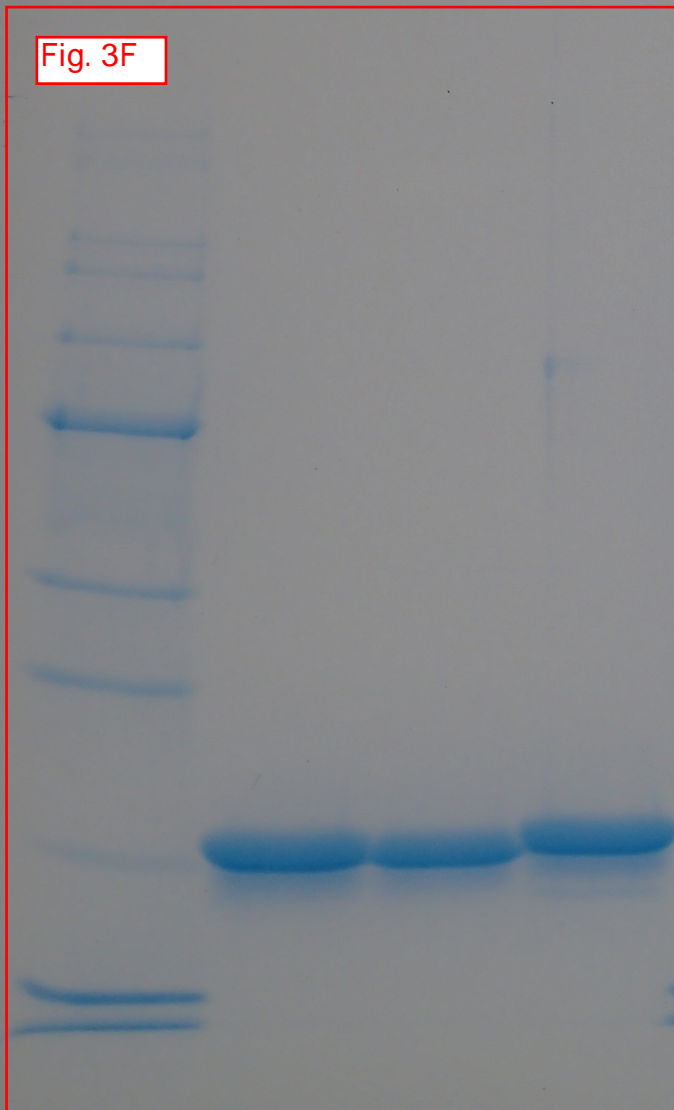

Supplement: S1 Raw images — (PDF) [file pone.0272992.s004.pdf]
