# Supplementary material for: Reverse mutants of the catalytic 19 kDa mutant protein (nanoKAZ/nanoLuc) from Oplophorus luciferase with coelenterazine as preferred substrate
Source: PLoS One. 2022 Sep 21;17(9):e0272992. doi: 10.1371/journal.pone.0272992 (PMC9491549; doi:10.1371/journal.pone.0272992)
Supplement: S1 Table — (DOC) [file pone.0272992.s002.doc]

**S1 Table. Primer list used for site-directed mutagenesis to prepare reverse mutant genes for nanoKAZ by PCR.**

| **Position** | **PCR step** | **Tempelate** | **Primer name** | | **Sequence** |
| --- | --- | --- | --- | --- | --- |
| **wK/nK-90I** | 1st | pcDNA3-GLsp-WNanoKAZ | a  b | nanoKAZ:V90I-F  nanoKAZ-3C/XbaI | 5’ gcc GTC GAC GAC CAC CAC TTC AAG ATT ATC CTG CAC TAC 3’  5’ gcc TCT AGA TTA GGC CAG GAT TCT CTC GCA CAG TCT 3’ |
| **wK/nK-115P** | 1st | pcDNA3-GLsp-WNanoKAZ | a  b | KAZ-8N/EcoRI  nanoKAZ:E115P-R | 5’ gcg GAA TTC TTT ACG TTG GCA GAT TTC GTT GGA 3’  5’ GAC GGC GAT GCC AGG GTA GGG TCT ACC 3’ |
|  |  | pcDNA3-GLsp-WNanoKAZ | c  d | nanoKAZ:E115P-F  nanoKAZ-3C/XbaI | 5’ GGT AGA CCC TAC CCT GGC ATC GCC GTC 3’  5’ gcc TCT AGA TTA GGC CAG GAT TCT CTC GCA CAG TCT 3’ |
|  | 2nd | 1st PCR  product | a  d | KAZ-8N/EcoRI  nanoKAZ-3C/XbaI | 5’ gcg GAA TTC TTT ACG TTG GCA GAT TTC GTT GGA 3’  5’ gcc TCT AGA TTA GGC CAG GAT TCT CTC GCA CAG TCT 3’ |
| **wK/nK-124Q** | 1st | pcDNA3-GLsp-WNanoKAZ | a  b | KAZ-8N/EcoRI  nanoKAZ:K124Q-R | 5’ gcg GAA TTC TTT ACG TTG GCA GAT TTC GTT GGA 3’  5’ GGT GAC GGT GAT CTG CTT GCC GTC GAA 3’ |
|  |  | pcDNA3-GLsp-WNanoKAZ | c  d | nanoKAZ:K124Q-F  BGH-R | 5’ TTC GAC GGC AAG CAG ATC ACC GTC ACC 3’  5’ TAG AAG GCA CAG TCC AGG 3’ |
|  | 2nd | 1st PCR product | a  d | KAZ-8N/EcoRI  nanoKAZ-3C/XbaI | 5’ gcg GAA TTC TTT ACG TTG GCA GAT TTC GTT GGA 3’  5’ gcc TCT AGA TTA GGC CAG GAT TCT CTC GCA CAG TCT 3’ |
| **wK/nK-138Y** | 1st | pcDNA3-GLsp-WNanoKAZ | a  b | KAZ-8N/EcoRI  nanoKAZ:I138Y-R | 5’ gcg GAA TTC TTT ACG TTG GCA GAT TTC GTT GGA 3’  5’ CAG TCT CTC GTC ATA GAT CTT GTT GCC 3’ |
|  |  | pcDNA3-GLsp-WNanoKAZ | c  d | nanoKAZ:I138Y-F  BGH-R | 5’ GGC AAC AAG ATC TAT GAC GAG AGA CTG 3’  5’ TAG AAG GCA CAG TCC AGG 3’ |
|  | 2nd | 1st PCR product | a  d | KAZ-8N/EcoRI  BGH-R | 5’ gcg GAA TTC TTT ACG TTG GCA GAT TTC GTT GGA 3’  5’ TAG AAG GCA CAG TCC AGG 3’ |
| **wK/nK-166N** | 1st | pcDNA3-GLsp-WNanoKAZ | a  b | KAZ-8N/EcoRI nanoKAZ:R166N-R | 5’ gcg GAA TTC TTT ACG TTG GCA GAT TTC GTT GGA 3’  5’ gcc TCT AGA TTA GGC CAG GAT GTT CTC GCA CAG TCT 3’ |
| **nK-AQLL** | 1st | pcDNA3-GLsp-nanoKAZ | a  b | T7  nKAZ-2R/AQQL | 5' TAA TAC GAC TCA CTA TAG GG 3'  5' TTC ACC CTG GCA GAC TTC GTC GGC GAC TGG CAA CAG ACC GCC GGC TAC AAC CAA GAC CAG GTC CTG GAG CAG GGC GGC TTG AGC AGC CTG TTC 3' |
|  |  | pcDNA3-GLsp-nanoKAZ | c  d | nKAZ-1F/AQQL  BGH-R | 5' TTC ACC CTG GCA GAC TTC GTC GGC GAC TGG CAA CAG ACC GCC GGC TAC AAC CAA GAC CAG GTC CTG GAG CAG GGC GGC TTG AGC AGC CTG TTC 3'  5’ TAG AAG GCA CAG TCG AGG 3’ |
|  | 2nd | 1st PCR  product | a  d | T7  BGH-R | 5' TAA TAC GAC TCA CTA TAG GG 3'  5’ TAG AAG GCA CAG TCG AGG 3’ |
| **nK-KVA** | 1st | pcDNA3-GLsp-nanoKAZ | a  b | T7  nKAZ-2R/AQQL | 5' TAA TAC GAC TCA CTA TAG GG 3'  5' CAG GCT GCT CAA GCC GCC CTG CTC CAG GAC CTG GTC TTG GTT GTA GCC GGC GGT CTG TTG CCA GTC GCC GAC GAA GTC TGC CAG GGT GAA GAC 3' |
|  |  |  |  |  |  |
|  |  | pcDNA3-GLsp-nanoKAZ | c  d | nKAZ-1F/AQQL  BGH-R | 5' TTC ACC CTG GCA GAC TTC GTC GGC GAC TGG CAA CAG ACC GCC GGC TAC AAC CAA GAC CAG GTC CTG GAG CAG GGC GGC TTG AGC AGC CTG TTC 3'  5' TAG AAG GCA CAG TCG AGG 3' |
|  |  |  |  |  |  |
|  | 2nd | 1st PCR  product | a  d | T7  BGH-R | 5' TAA TAC GAC TCA CTA TAG GG 3'  5’ TAG AAG GCA CAG TCG AGG 3’ |
|  |  |  |  |  |  |
| **nK-FLM** | 1st | pcDNA3-GLsp-nanoKAZ | a  b | T7  nKAZ-6R/FLM | 5' TAA TAC GAC TCA CTA TAG GG 3'  5' CTT GAA GAT CAT CTC GAT TAG GCC CAT CTG AAA GCC GCT CAG GCC 3' |
|  |  |  |  |  |  |
|  |  | pcDNA3-GLsp-nanoKAZ | c  d | nKAZ-5F/FLM  BGH-R | 5' CTG AGC GGC TTT CAG ATG GGC CTA ATC GAG ATG ATC TTC AAG GTC 3'  5' TAG AAG GCA CAG TCG AGG 3' |
|  |  |  |  |  |  |
|  | 2nd | 1st PCR  product | a  d | T7  BGH-R | 5' TAA TAC GAC TCA CTA TAG GG 3'  5’ TAG AAG GCA CAG TCG AGG 3’ |
| **nK-4A** | 1st | pcDNA3-GLsp-nanoKAZ | a  b | E4A-nKAZ  BGH-R | 5' ccg GAA TTC TTC ACC CTG GCC GAC TTC GTC GGC GAC TGG 3’  5’ TAG AAG GCA CAG TCG AGG 3’ |
| **nK-11Q** | 1st | pcDNA3-GLsp-nanoKAZ | a  b | R11Q-nKAZ  BGH-R | 5' ccg GAA TTC TTC ACC CTG GAG GAC TTC GTC GGC GAC TGG CAG CAG ACC GCC GGC TAC 3'  5’ TAG AAG GCA CAG TCG AGG 3’ |
| **nK-18Q** | 1st | pcDNA3-GLsp-nanoKAZ | a  b | L18Q-nKAZ  BGH-R  V27L-nK  BGH-R | 5' ccg GAA TTC TTC ACC CTG GAG GAC TTC GTC GGC GAC TGG AGA CAG ACC GCC GGC TAC AAC CAG GAC CAG GTC CTG GAG 3'  5’ TAG AAG GCA CAG TCG AGG 3’  5' ccg GAA TTC TTC ACC CTG GAG GAC TTC GTC GGC GAC TGG AGA CAG ACC GCC GGC TAC AAC CTG GAC CAG GTC CTG GAG CAG GGC GGC CTG AGC AGC CTG T 3'  5’ TAG AAG GCA CAG TCG AGG 3’ |
| **nK-27L** | 1st | pcDNA3-GLsp-nanoKAZ | a  b |  |  |
| **nK-4A11Q** | 1st | nK-11Q | a  b | E4A-nKAZ  BGH-R | 5' ccg GAA TTC TTC ACC CTG GCC GAC TTC GTC GGC GAC TGG 3’  5’ TAG AAG GCA CAG TCG AGG 3’ |
| **nK-4A18Q** | 1st | nK-L18Q | a  b | E4A-nKAZ  BGH-R | 5' ccg GAA TTC TTC ACC CTG GCC GAC TTC GTC GGC GAC TGG 3’  5’ TAG AAG GCA CAG TCG AGG 3’ |
| **nK-4A27L** | 1st | nK-V27L | a  b | E4A-nKAZ  BGH-R | 5' ccg GAA TTC TTC ACC CTG GCC GAC TTC GTC GGC GAC TGG 3’  5’ TAG AAG GCA CAG TCG AGG 3’ |
| **nK-11Q18Q** | 1st | nK-L18Q | a  b | R11Q-nKAZ  BGH-R | 5' ccg GAA TTC TTC ACC CTG GAG GAC TTC GTC GGC GAC TGG CAG CAG ACC GCC GGC TAC 3'  5’ TAG AAG GCA CAG TCG AGG 3’ |
| **nK-11Q27L** | 1st | nK-V27L | a  b | R11Q-nKAZ  BGH-R | 5' ccg GAA TTC TTC ACC CTG GAG GAC TTC GTC GGC GAC TGG CAG CAG ACC GCC GGC TAC 3'  5’ TAG AAG GCA CAG TCG AGG 3’ |
| **nK-18Q27L** | 1st | nK-V27L | a  b | L18Q-nKAZ  BGH-R | 5' ccg GAA TTC TTC ACC CTG GAG GAC TTC GTC GGC GAC TGG AGA CAG ACC GCC GGC TAC AAC CAG GAC CAG GTC CTG GAG 3'  5’ TAG AAG GCA CAG TCG AGG 3’ |
