# Supplementary material for: Reverse mutants of the catalytic 19 kDa mutant protein (nanoKAZ/nanoLuc) from Oplophorus luciferase with coelenterazine as preferred substrate
Source: PLoS One. 2022 Sep 21;17(9):e0272992. doi: 10.1371/journal.pone.0272992 (PMC9491549; doi:10.1371/journal.pone.0272992)
Supplement: S2 Table — (DOC) [file pone.0272992.s003.doc]

**S2 Table. Purification of QL-nanoKAZ from 800 mL of cultured *E. coli* cells using a Ni-chelate column.**

| Steps | Total volume  （mL） | Total protein  （mg） (%) | Total activity ^a^  (*I*_max_, ×10^11^ rlu) (%) | Specific activity  (×10^9^ rlu/mg） |
| --- | --- | --- | --- | --- |
|
| 1) 1st Ni-chelate column (ø2.5 × 5 cm) | 60 | 466.3 (100) | 29.9 (100) | 6.4 |
| Eluted fractions with 0.1 M imidazole | 18 | 154.2 (33) | 23.7 (79) | 15.4 |
| 2) 2nd Ni-chelate column (ø1.5 × 6 cm)  (Diluted fractions of 1st Ni-chelate column eluent) | 200 | 98.8 (100) | 28.8 (100) | 23.1 |
| Eluted fractions with 0.1 M imidazole | 18 | 91.8 (93) | 19.9 (69) | 21.7 |

^a^ Luminescence activity was determined using an AB2200 luminometer with a 0.23% neutral density filter.
